# Implementation of neurological group-based telerehabilitation within existing healthcare during the COVID-19 pandemic: a mixed methods evaluation

**DOI:** 10.1186/s12913-023-09635-w

**Published:** 2023-06-21

**Authors:** Suzanne Ackerley, Neil Wilson, Paul Boland, Jessica Read, Louise Connell

**Affiliations:** 1 School of Sport and Health Sciences, University of Central Lancashire, Preston, Lancashire UK; 2 East Lancashire Hospitals NHS Trust, Burnley, Lancashire UK; 3 Lancashire & South Cumbria NHS Foundation Trust, Blackburn, Lancashire UK

**Keywords:** Telerehabilitation, Brain injury, Stroke, Neurological, Rehabilitation, Mixed methods

## Abstract

**Background:**

There is a need to evaluate if and how telerehabilitation approaches might co-exist within healthcare in the long-term. Our aim was to implement and evaluate a multidisciplinary group-based telerehabilitation approach for people engaging in neurological rehabilitation.

**Methods:**

NeuroRehabilitation OnLine (NROL) was adapted and implemented within an existing healthcare system as a programme of repeating six-week blocks. A robust evaluation was undertaken simultaneously using a convergent parallel design underpinned by implementation frameworks. This included service data, and patient and staff interviews. Implementation success was conceptualised using the outcomes of appropriateness, acceptability and sustainability.

**Results:**

Eight NROL blocks delivered 265 sessions with 1347 patient contacts, and NROL continues as part of standard practice. The approach was appropriate for varied demographics and had positive patient opinions and outcomes for many. Staff perceived NROL provided a compatible means to increase therapy and help meet targets, despite needing to mitigate some challenges when fitting the approach within the existing system. NROL was considered acceptable due to good attendance (68%), low drop-out (12%), and a good safety record (one non-injury fall). It was accepted as a new way of working across rehabilitation disciplines as an ‘extra layer of therapy’. NROL had perceived advantages in terms of patient and staff resource (e.g. saving time, energy and travel). NROL provided staffing efficiencies (ratio 0.6) compared to one-to-one delivery. Technology difficulties and reluctance were surmountable with dedicated technology assistance. Leadership commitment was considered key to enable the efforts needed for implementation and sustained use.

**Conclusion:**

Pragmatic implementation of group-based telerehabilitation was possible as an adjunct to neurological rehabilitation within an existing healthcare system. The compelling advantages reported of having NROL as part of rehabilitation supports the continued use of this telerehabilitation approach. This project provides an exemplar of how evaluation can be run concurrently with implementation, applying a data driven rather than anecdotal approach to implementation.

**Supplementary Information:**

The online version contains supplementary material available at 10.1186/s12913-023-09635-w.

## Background

The ambition to harness the potential of technology to transform healthcare has been expressed in policy for years [1, 2]. Although telerehabilitation has existed in some countries for several years [3, 4] overall adoption has been slow [5, 6], and the pandemic provided the impetus for a dramatic increase in uptake of telehealth [7]. Globally rehabilitation services faced substantial disruptions with variable provision of in-person therapy, group therapies suspended, and diminished workforce capacity [8–11]. In many healthcare services, telerehabilitation was readily instigated into routine practice to provide timely care to the substantial and increasing number of service users requiring rehabilitation [8]. This hastened approach to implementation was warranted within the context of the pandemic, but now there is a need to robustly evaluate if and how telerehabilitation approaches might co-exist within healthcare systems in the long-term [12, 13].

Telerehabilitation involves the provision of one or more rehabilitation disciplines delivered remotely via telecommunication devices. It can be used as an alternative delivery method for providing conventional in-person rehabilitation. Within neurological rehabilitation it has been used for delivery of upper and lower limb training, mobility training, post-hospital discharge support programs and communication therapies [14–17]. Cochrane reviews have shown low to moderate quality evidence that telerehabilitation is as effective as in-person rehabilitation for Stroke and Multiple Sclerosis (MS) patients [16, 17]. Positive findings are also emerging from data collected during the rapid expanded use of telerehabilitation during the pandemic [13, 18]. Looking forward, telerehabilitation may contribute to a hybrid approach for delivering rehabilitation services [13, 19–21]. Some challenges are likely to exist when integrating and sustaining telerehabilitation within healthcare practice such as the requirement for dedicated resources and support, adequate environment, and training [22, 23]. Nevertheless, telerehabilitation has the potential to facilitate timely and equitable rehabilitation by mitigating some barriers to access and provision, but it is important to ensure that patient and service outcomes are comprehensively evaluated [13, 21].

A promising multi-disciplinary group-based telerehabilitation approach called Neuro-Rehabilitation OnLine (N-ROL) was developed in London in response to the first lockdown in the UK [24]. They delivered a standalone 15-week programme to predominantly stroke patients. Their programme consisted of physical and talking therapy groups set within a holistic working approach, incorporating psychoeducation and peer-support elements (for full TIDieR description see Beare and colleagues) [24]. Results were positive with participating patients improving significantly on patient outcome measures. The aim of the current project was to adapt and implement a tailored version of NROL to see how it could be delivered when embedded within an existing healthcare system. Robust evaluation was undertaken alongside implementation to determine the appropriateness, acceptability, and to provide direction for sustained use.

## Methods

### Implementation context and setting

This project is a collaboration between a healthcare organisation (East Lancashire Hospitals NHS Trust, ELHT) and researchers (University of Central Lancashire, UCLan), as part of an established clinical academic partnership. It is registered with ELHT as a service evaluation and for the qualitative study approval has been given by UCLan Health Ethics Review Panel (HEALTH 0155).

The healthcare setting is a neurological rehabilitation service consisting of two teams, the Neurorehabilitation team and Stroke therapy team. The teams provide multi-disciplinary rehabilitation across hospital and community settings to adults who have a sudden onset or progressive/intermittent neurological condition. The service typically offers in-person individual and group therapy. During the evaluation period (January 2021—April 2022) in-person group therapies were suspended and individual in-person inputs were intermittently curtailed due to pandemic restrictions. From January 2021, patients undertaking outpatient or community neurological rehabilitation could be referred by a treating therapy staff member to participate in NROL as an adjunct to their existing rehabilitation. In addition to service eligibility, inclusion to NROL required patients to be English speaking or have access to a translator and have willingness to participate in online group therapy with or without carer support. Patients were excluded from NROL if they lacked access to appropriate computer hardware or connectivity.

### NROL

NROL was adapted for purpose from the standalone version of N-ROL described by Beare and colleagues [24]. In brief, this updated version of NROL delivers synchronous (in real-time) group-based neurological rehabilitation to outpatient and community patients over six-week recurring blocks via the online platform Microsoft Teams. It consists of talking (Cognitive education, Cognitive processing, Living Well, Fatigue, Dysarthria, Dysphasia and Bookclub) and physical (Balance & Mobility and Upper Limb) targeted therapy groups and community groups (NROL entry, exit, follow-up and Café NROL). Groups are run by a multidisciplinary team comprising therapy staff (allied health professional and psychology), assistant practitioners and patient volunteers. NROL, and its groups, are described in more detail elsewhere [25–31]. To facilitate access to NROL patients are provided with set-up and ongoing technical assistance by a dedicated NROL technology staff member. Technology assistance is also available for staff and patients during all NROL sessions. Administrative support is provided by a service administrator within the scope of their role. Oversight for NROL is provided by project leads and a coordinator.

### Design

**Fig. 1:**
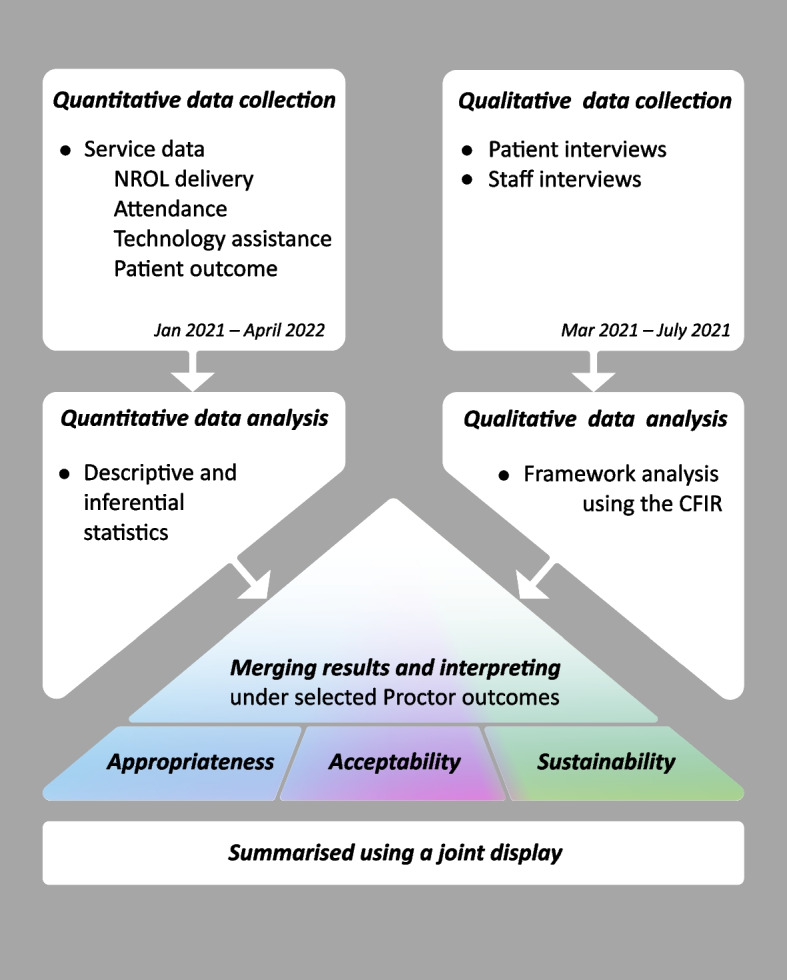
Mixed methods convergent parallel design

A convergent parallel design was used throughout delivery of NROL including data collection and analysis at a service level, and patient and staff interviews. Both quantitative and qualitative data were collected, analysed separately, then merged and interpreted together. Implementation success was conceptualised using selected Proctor outcomes [32]. These were chosen to fit the evaluation purpose and agreed with decision-makers (commissioners and managers) as those that were relevant for our evaluation, informed by the early stage of implementation and resources available. The outcomes were appropriateness (perceived fit, relevance, or compatibility), acceptability (attendance, safety and perception among implementation stakeholders) and sustainability (key considerations for NROL to continue as a routine part of care delivery) (Fig. 1).

## Quantitative data collection and analysis

Service data included details of NROL delivery (number of blocks, groups, and sessions), patient and staff attendance, technology assistance provided, and patient outcome.

Three patient-reported outcome measures were used on entry and exit to NROL. The Stroke Self-Efficacy Questionnaire (SSEQ, max 39) provided a self-report measure of self-efficacy [33, 34]. Modification of SSEQ wording was required to be inclusive of ‘stroke and other neurological conditions’. The EQ-5D-5L provided measures of health-related quality of life [35]. It consists of 2 parts: the EQ Visual Analogue Scale (EQ-VAS, max 100) rates individual’s perceived overall current health and the EQ descriptive system profiles individual’s health state. The descriptive system question responses were transformed into an EQ Index score (EQ-Index, max 1.000) using the EuroQoL Group’s crosswalk methodology with a United Kingdom population value set [36]. The Patient Specific Functional Scale (PSFS, max 10) was used to generate patients’ average rating on performing activities of function that are important to them and which were impacted by their neurological condition [37, 38].

All patients referred to NROL were characterised using routinely collected demographic and clinical data and analysed. Reasons for withdrawal or drop-out from NROL were recorded. Intention to treat analysis was used for patients who dropped out of NROL after participating in at least one targeted therapy session, or when patients withdrew from a specific group.

Descriptive statistics were used to summarise service level data. For patient outcome data, because some outcomes were not normally distributed and had outliers, we report non-parametric tests for all to allow a more conservative test of significance. Wilcoxon signed rank tests were used to test for change from entry to exit. Mean differences are provided for comparison with minimal clinically important differences (MCIDs), defined as SSEQ ≥ 4 points, EQ-VAS ≥ 10 points, EQ-Index ≥ 0.080 and PSFS ≥ 3 points using published data or 10% of maximum total score [38–40]. The proportion of patients who improved or remained stable (change score (exit – entry) ≥ 0), and those who met or exceeded the MCID, are reported. Subgroup analyses were conducted to help understand the appropriateness of NROL to different patient cohorts given the varied demographics, which included patients with conditions with differing expected disability trajectories and time periods since diagnosis. Chi-Square Tests, or where appropriate Fisher’s Exact Test 2 × 2 comparisons, were used to determine any association between the direction of change in patient-reported outcome measure score (improved or stable (change score ≥ 0) vs. declined (change score < 0)) and condition category (sudden onset vs. progressive/intermittent) or chronicity (subacute vs. chronic). Classification of conditions was guided by the NHS Long Term Conditions National Service Framework [41], and chronicity defined as subacute if 1 week to six months after diagnosis and chronic if over six months [42].

Analyses were performed using Microsoft Excel 2008 and IBM SPSS Statistics version 28.0. Statistical significance was set at α < 0.05 and all tests were two-tailed. For multiple comparisons we used the Benjamini–Hochberg procedure, with the false discovery rate fixed to 5% [43].

## Qualitative data collection and analysis

Patients and healthcare staff were invited to participate in a one-off interview if they had used or been involved with NROL. A purposive approach ensured a broad mix of participants in terms of gender, age, ethnicity, diagnosis (patients) and profession and job role (staff).

The Consolidated Framework for Implementation Research (CFIR) [44] was used in the development of the interview guide for the study, based on a previous interview guide used to evaluate the implementation of stroke rehabilitation interventions [45–47] (see detail in the supplementary file: interview guide). The CFIR was chosen as it is one of the most commonly used determinant frameworks and provides a menu of constructs that have been associated with effective implementation [44, 48]. Domains include characteristics of the individuals, intervention, and inner setting. Interviews were conducted over Microsoft Teams and lasted approximately 30 and 60 min. Participants were aware that the interviewer was not part of the clinical team, and an honest perspective was wanted to learn lessons for implementation, and that criticisms were welcomed. Interviews were recorded, anonymized, and transcribed.

### Researcher characteristics and reflexivity

This project was part of a clinical academic partnership, with LC & SA being both experienced researchers and physiotherapists in neurological rehabilitation. JR is a psychologist who works in the Neurorehabilitation team and was involved in design and delivery of NROL content. Two further researchers (NW & PB) were involved in the analysis and interpretation of the data and were not from a clinical background and independent from the clinical team to help reduce any associated bias from those associated with implementing the NROL intervention. NW is a researcher with a social psychology background and PB is experienced in health services research.

### Data analysis

Interview transcripts were imported into NVivo 12 for analysis. Framework analysis was undertaken using the CFIR to code data deductively, with additional free codes developed where needed. To establish a shared understanding and interpretation of the coding framework, all researchers started by coding the same two transcripts. The coded transcripts were compared and any variance in interpretation of data and application of codes was discussed to arrive at a mutual decision. Subsequently the remaining transcripts were coded separately by two researchers independently (with one from a clinical background and one from an external research perspective). The relevant CFIR domains to this evaluation (characteristics of NROL, the individuals (staff and patients) and the inner setting (Neurorehabilitation and Stroke teams) relating to the implementation outcomes are reported.

## Mixed methods integration

A joint display, defined as a way to “integrate the data together through a visual means to draw out new insights beyond the information gained from the separate quantitative and qualitative results.” [49] provided the structure to integrate quantitative and qualitative analyses to explore the implementation outcomes of appropriateness, acceptability and sustainability. This is a suggested method to integrate and represent mixed methods analyses [50]. The juxtaposing of quantitative results and qualitative findings side-by-side facilitated insights in relation to the implementation outcomes. These were discussed and agreed by the research team and the clinical staff involved in NROL.

## Results

### NROL participation

A total of 137 patients were referred within the evaluation period (January 2021 – April 2022). To understand NROL fit with the overall service, a small-scale review was made over the period from January 2021 to August 2021 between patients referred to NROL and those who were not. This indicated NROL referrals were received for about 10% of patients receiving outpatient or community neurological rehabilitation services. Evaluation of selected demographics (age, sex, index multiple deprivation) indicated patients referred to NROL were younger (*Mdn* = 54y, IQR = 43.5–65) than those not referred (*Mdn* = 67y, IQR = 54–77), *p* < 0.001).

**Fig. 2:**
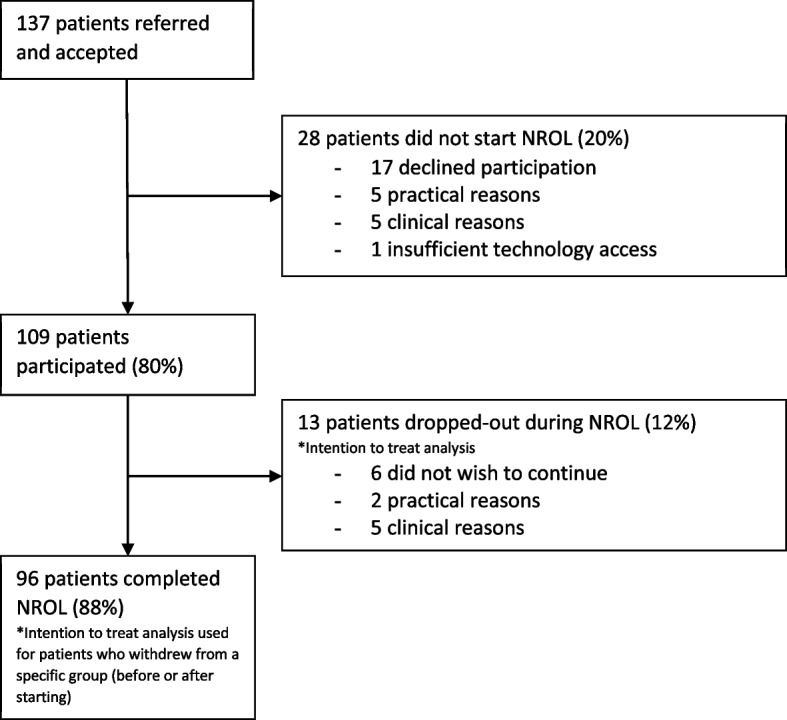
NROL participation profile

All NROL referrals were accepted; 109 patients participated in NROL, 28 patients did not start for a variety of reasons (Fig. 2). Participating patient characteristics are presented in Table 1 and are comparable with those who were referred to NROL but did not start (all *p* > 0.05).

**Table 1:** Patient demographics

Demographic	Participated (*n* = 109)
Age, median (range)	57 (19 – 86)
Sex, male (%)	66 (61%)
Ethnicity	
White	76 (70%)
Asian or Asian British	10 (9%)
Not reported	23 (21%)
Index multiple deprivation, median (range)	4 (1 – 10)
Living alone, yes (%)	14 (13%)
Condition	
Sudden	80 (73%)
Stroke	48 (44%)
Trauma	12 (11%)
Other acquired	15 (14%)
Peripheral nervous system injury	4 (4%)
Spinal cord injury	1 (1%)
Progressive/intermittent	29 (27%)
Multiple Sclerosis	17 (16%)
Other	12 (11%)
Chronicity	
Subacute	49 (45%)
Chronic (6 m +)	60 (55%)

### Service data

#### NROL delivery

Eight blocks of NROL were undertaken in the evaluation period. The median number of patients participating in a block was 21 (range = 12–26). 13 different groups were offered across a range of therapies, with the median number of groups per block being 8 (range = 6–9). Groups offered and session content was iteratively modified to meet the requirements of NROL patient cohorts. In total, 265 NROL sessions were delivered with a median of 4 patients per session (range = 1–14). Summary data including individual group sessions delivered are presented in Table 2. One adverse event was recorded: a non-injury fall in a physical group. NROL staff followed standard operating procedure guidelines for managing and documenting this event, no first aid or follow-up was required. NROL continues to be delivered successfully beyond the evaluation period and has become part of standard care.

**Table 2:** Service data summary over 8 blocks of NROL

Group	Sessions delivered, n	Patients participated, n	Proportion patients in group, %	Patient contacts	Patient session attendance rate, %	Staff contacts	Staff:patient ratio, median (range)
**Total (incl. Café NROL)**	265	109	100%	1347	59%	727	0.6 (0.1 – 4.0)
**Total (excl. Café NROL)**	229	109	100%	1053	68%	628	0.7 (0.1 – 4.0)
** Talking (Targeted therapy)**	118	82	75%	511	71%	321	0.8 (0.1 – 4.0)
Cognitive education	43	34	31%	172	74%	135	0.8 (0.4 – 3.0)
Cognitive processing	10	8	7%	26	70%	30	1.3 (0.6 – 4.0)
Living Well (adjustment/wellbeing)	23	51	47%	115	66%	62	0.5 (0.3 – 2.0)
Fatigue	12	20	18%	91	67%	29	0.3 (0.1 – 0.5)
Dysarthria group	15	9	8%	56	77%	33	0.5 (0.4 – 1.3)
Dysphasia group	10	6	6%	28	93%	15	0.5 (0.3 – 2.0)
Bookclub (information processing)	5	6	6%	23	66%	17	0.8 (0.6 – 1.0)
** Physical (Targeted therapy)**	91	62	57%	393	69%	249	0.7 (0.3 – 3.0)
Balance & Mobility group	67	53	49%	325	71%	189	0.6 (0.3 – 1.5)
Upper Limb group	24	14	13%	68	60%	60	1.0 (0.3 – 3.0)
** Community (excl. Café NROL)**	20	88	81%	149	59%	58	0.4 (0.2 – 3.0)
NROL entry	11	82	75%	84	87%	36	0.5 (0.2 – 3.0)
NROL exit	8	42	39%	59	38%	20	0.3 (0.2 – 0.5)
NROL follow-up	1	6	6%	6	86%	2	0.3 (0.3)
** Community: Café NROL** (discussion/peer-support)	36	68	62%	294	40%	99	0.3 (0.2 – 1.0)

#### Patient attendance

Most patients participated in 1 NROL block (55%), but some participated in 2 (36%), 3 (7%) or 4 (2%). There were 1347 patient contacts in total. Overall NROL attendance rate was 68% (excluding the Café NROL group which was optional to attend). Attendance rate at targeted therapy groups (talking and physical) ranged from 93 to 60%. Sixty-eight patients (62%) chose to participate in Café NROL. Summary data including individual group attendance are presented in Table 2.

#### Staff attendance

There were 727 staff contacts (including therapy staff, assistant practitioner and technology support) during delivery of the 1347 patient contacts. This included staff attendance for observation/training purposes. The median staff:patient ratio across all sessions was 0.6 (range = 0.1 – 4.0). All groups were delivered with a median staff:patient ratio less than or equal to 1.0, except the Cognitive processing group (*Mdn* = 1.3). The talking groups had the highest staff:patient ratio (*Mdn* = 0.8, range = 0.1 – 4.0) and the community groups the lowest (*Mdn* = 0.4, range = 0.2 – 3.0). The occurrence of staff:patient ratios with values up to 4.0, as indicated by the reported ranges, reflects the fact that sessions were required to have a minimum number of staff attending regardless of the number of patients present and that many sessions included staff for experiential learning. Summary data for individual group contacts and staff:patient ratios are presented in Table 2. There were 84 student and 34 patient-volunteer contacts.

#### Technology assistance

Total technology assistance for set-up and any further individual support (excluding in-session technology assistance which is captured in staff:patient ratio) took a median of 11 min (IQR = 6–22, max 250 min) and 1 contact (IQR = 1–2, max 9) per patient. The main technology areas addressed included: assisting installation of Teams software, advising on use of the Teams App (rather than the Browser due to limited functionality), aiding use of microphone and camera functions, enabling patients to see other group members (rather than only the person speaking), and building a patient’s overall skill and confidence to enable successful use of Teams and its access through email invites. Technology assistance was offered to carers if required.

#### Patient outcome data

Complete data sets were available for SSEQ, EQ-5D-5L and PSFS for 84, 96 and 78 patients, respectively. Variation in number is largely due to phased introduction of these measures during pragmatic implementation.

At a group level, statistically significant improvements were seen over time in all patient outcome measures, with all *p*-values significant using the Benjamini–Hochberg procedure with our false discovery rate (SSEQ: *Mdn* entry = 19 (IQR = 14–23.75), *Mdn* exit = 21 (IQR = 14–25), p = 0.027 (*M* difference = 1.50 (SD = 6.37)); EQ-Index: *Mdn* entry = 0.547 (IQR = 0.231–0.663), *Mdn* exit = 0.558 (IQR = 0.418–0.691), *p* = 0.001 (*M* difference = 0.083 (SD = 0.228); EQ-VAS score: *Mdn* entry = 50 (IQR = 40–70), *Mdn* exit = 60 (IQR = 45 – 75), *p* = 0.022 (*M* difference = 3.80 (SD = 21.52)); PSFS: *Mdn* entry = 3.5 (IQR = 2.0–4.5), *Mdn* exit = 5.5 (IQR = 3.50–7.13), *p* < 0.001 (*M* difference = 1.98 (SD = 2.09)). Large overlaps in interquartile ranges (and standard deviations) are noted and only EQ-Index mean difference met the MCID.

At an individual level, the proportions of patients who improved or remained stable were between 62%—86% (SSEQ: 62%; EQ-Index: 67%; EQ-VAS: 68%; PSFS: 86%), with 36%—49% (SSEQ: 38%; EQ-Index: 41%; EQ-VAS: 49%; PSFS: 36%) of patients meeting or exceeding the MCID.

#### Subgroup analyses

Subgroup analyses indicated no association between the direction of change in SSEQ, EQ-VAS or EQ-Index, or PSFS scores (improved or stable vs. declined) and condition (sudden vs. progressive/intermittent) or chronicity (subacute vs. chronic) (all *p* > 0.05).

### Qualitative data

Seventeen staff and 13 patient interviews were conducted between March- July 2021. Demographics are detailed in Table 3.

**Table 3:** Interviewed patient and staff demographics

Patient demographics	Interviewed patients (*n* = 13)	Staff demographics	Interviewed staff (*n* = 17)
Age		Sex	
Mean age (range)	53 (25 – 78)	Male	4 (24%)
Sex		Discipline	
Male	5 (39%)	Occupational Therapists	6 (35%)
Ethnicity		Physiotherapists	5 (29%)
White	6 (46%)	Psychologist	1 (6%)
Asian or Asian British	1 (8%)	Speech Language Therapists	3 (18%)
Unreported	6 (46%)	Assistant Practitioner	1 (6%)
Index multiple deprivation		Technology Support	1 (6%)
Median IMD (range)	6 (2 – 9)	Team	
Living alone		Neurorehabilitation team	7 (41%)
Yes	2 (15%)	Stroke therapy team	5 (29%)
Condition		Both teams	5 (29%)
Sudden	8 (62%)	Seniority	
Progressive/intermittent	5 (39%)	Band 4	1 (6%)
Chronicity		Band 5	1 (6%)
Subacute	1 (8%)	Band 6	4 (24%)
Chronic (6 m +)	12 (92%)	Band 7	7 (41%)
		Band 8/ Management	2 (12%)
		Student	1 (6%)

## Constructs influencing implementation of NROL

Constructs that were reported as key to the implementation of NROL are summarised in Tables 4, 5 and 6 according to the relevant CFIR domains, together with exemplar quotes. Further detail is available in a Supplementary file: interview analysis. Participants are identified by their participant code. For the ‘characteristics of the individuals’ and ‘characteristics of NROL’ domains, both staff and patients highlighted issues related to the implementation outcomes. For the ‘inner setting’, only staff quotes are given as patients were generally unaware of the constructs related to the service itself.

**Table 4:** Key characteristics of individuals and exemplar quotes

**Construct**		**Summary**	**Exemplar quotes**
**Knowledge and beliefs**	Staff	Generally positive opinions of NROL	*S07:* We're getting a lot of staff that feel it's worthwhile. A lot of patients that feel it's worthwhile as well, and a lot of really positive feedback from the staff that are doing the sessions.
		NROL was valuable for increasing opportunity for practice	*S11:* Provided a lot of opportunities for practice, and to learn new skills and even remind them of some of what they've already gone over…
		Some reluctance to changing practice	*S07:* NROL has its place and there are a lot of benefits to NROL, but I think as a team we need to make sure that we're not diluting our service for our face-to-face contact. *S02:* Some staff just don't wanna do it. Some people don't want the tech. Some people don't want to change their practice. Some people say it's too much hassle, and they can just go along with what they know. And so I think there's a whole raft of reasons, some of which are acceptable and some of which aren't.
	Patients	Positive experience of NROL from a physical and mental health perspective	*P03:* ending up after the exercise session in a complete sweat and absolutely knackered [Laughter]. Which is always a good sign. And they can see what you're doing and what the issues are and they can give you advice on how to correct. I didn't think they could do that through a video link, but they can, they absolutely can. *P05:* it meant so much. Because between appointments, you tend to feel abandoned nobody is listening, nobody is bothered by what problems you have, and this a way of discussing things. So for me, it's just been a great benefit…. I can’t praise the sessions enough basically.
		NROL provided an extra layer of therapy	*P01*: To me NROL is an extra addition to a person's normal therapy and it just provides an additional layer of therapy to a person.
		Group format with peer support identified as valuable	*P03:* You get a little bit of a bond with the people you're doing that with 'cause you're all going through a similar kind of thing, … I think seeing other people and how they are progressing. It gives you a little bit of motivation to progress yourself. You feel like you're not on your own. Sounds a bit stupid, but you know, sometimes you can feel you're battling this on your own and actually, you’re not.
**Self-efficacy**	Staff	Gained confidence over time	*S01:* the first block was quite nervy to be honest. 'Cause it was new … as I've done a few groups, I’m less anxious now. *S09:* I've enjoyed the different platform and the challenges. It's made me adapt as a clinician.
	Patients	Gained confidence and motivation	*P13:* before I was super confident, I wouldn’t have any problems …so this is giving me that bit of confidence to talk to people a bit more… You know, it's like a stepping stone I guess. *P02:* I had to quit work, I was self-employed. I could barely speak to the customers, … but now I've been interacting with people. It's made me able to interact more with strangers. It’s helping me come round. *P07:* I said I've got grade 8 on my piano,… And I said, I've not touched it…they were like ‘no come on, do it, do it do it’. Now, I’m playing the piano and I'm walking a mile into my local town. Yeah, whereas before I used to get taxi.

**Table 5:** Key characteristics of NROL and exemplar quotes

**Construct**		**Summary**	**Exemplar quotes**
**Complexity**	Staff	Effortful to initiate	S04: it was new, we had to develop our group… so that was a lot of work initially.
		Technology assistance was key	S12: they had the skills from the physio point of view. I'm not sure they did from the technical point of view. When [tech support] wasn't around it was more difficult
		Challenges were surmountable	S01: A lot of it was just trying to problem solve with many different devices trying to access the same platform… all reacting in different ways … in all cases there’s always been work around.
	Patients	Technology assistance was key	P01: The only thing that would make it difficult for people is the technology, lack of familiarity with it and physically getting on…. And that’s why you need a person like (technology support role) and, you have to clone him.
**Relative advantage**	Staff	Advantages in terms of resource, saving: - Time - Energy (physical & mental) - Travel	*S02:* For face-to-face groups… you need to find premises, you need to deal with the transport issues, you need to deal with the care issues, the practicalities of toileting and wheelchairs and drinks, …. so actually there's a reluctance sometimes to develop groups face-to-face, because of all the complexities. But this is an easy way of doing it. *S13:* in terms of efficiency to some extent, we're not having to travel to five different patients. We can see them all in one go. So it's good from that respect. … you're not claiming on expenses, from an eco friendly point of view, you're cutting down on your carbon emissions as well.
		Group format with peer support identified as valuable	*S08:* I think they’ve actually taken on board the education better because they’re hearing it from each other not just from a therapist who hasn’t experienced having a brain injury. *S03:* So if that peer support can give them the feeling of not being the only one… if that can help with their wellbeing…they're gonna feel more motivated to do the therapy…
	Patients	Advantages in terms of resource, saving: - Time, - Energy (physical & mental) - Travel	*P06:* you’re not tired when you arrive…I used to joke it's a full time job being ill… I use patient transport so it can be half a day to a day …Well, this is short sharp burst. It's easy, it's manageable, and you can do it. It's done and you can integrate it then into your life. If it's a speaking thing or it's a physical thing, you can recuperate faster and you can do some more.
		Group format with peer support identified as valuable	*P06:* I've always extolled the benefits of doing things within a group. You build a bit of camaraderie, you get shared learning and experiences. It's a good model to use. So there's the therapeutic effect… and we had fun learning and there was still a tangibility at the end of it. *P03:* it was actually really good to see how I was comparing against the other people… So you don't even get that kind of insight when you are in a 1:1 situation at a hospital.
**Adaptability**	Staff	Continuous adaptation required	*S14:* The group should meet the needs of the patients, not the patients meet the needs of the groups. We should constantly review what the groups are offering as to the patients that are referred, so making sure that it kind of tailors to their needs, not ours. *S03:* so far the majority of it is being run by, qualified therapists… a lot of them are band sixes and band sevens, which to get things off the ground and to make sure it's running well is, you know I can understand why … Once things are a bit more established, I think we need to play about with the experience levels … and utilize assistants a bit more…
	Patients	Modifiable to patient need	*P01:* …he’d [the physio] see some things I was struggling on… ‘perhaps just do it from sat down’…so they could modify, and I think that's the benefit as well. *P06:* We got the feeling, it's bespoke really. And to be fair a lot of this has felt bespoke which is nice and rare.

**Table 6:** Key characteristics of inner setting and exemplar quotes

**Construct**		**Summary**	**Exemplar quotes**
**Culture**	Staff	Understanding of ‘normal’ service provision to consider how NROL fits	*S03:* we have to be careful that it’s not… the icing on the cake therapy, that they are actually increasing the intensity of the overall offer. But then I think it comes back to what's our core business, and are we delivering our core business? *S14:* We don't provide maintenance, we provide a block of treatment to improve a patient and then discharge them. But then do we need to actually relook at the model?
		NROL requires a shift in culture	*S02:* To deliver this is requiring a culture shift and a mindset shift in the therapists, and they're not all ready for that yet. So we've only had a few groups running. There's resistance in some places, and there's absolutely enthusiasm in others. *S15:* It's just trying to sort of weave NROL into the daily fabric of life rather than feeling like a separate thing. So I think we're getting there, but it’s slow.
**Implementation Climate**	Staff	Compatibility of NROL within the service with competing priorities and limited capacity	*S13:* Capacity and the time are the main issues. I think it's been difficult. Trying to… I suppose juggling my daytime job and NROL. *S03:* NROL could be a way of reducing waiting lists… but I think we're in a situation where the therapists are so busy they can't see the wood from the trees. S17: it's been a year of stress at work and outside of work for many reasons…during COVID the stroke team actually started a new service, the early intervention team….so there's just been a mass of things going on, so I think people have just not had the headspace for something else.
		NROL enabled collaborative working across teams, caseloads, disciplines	*S08:* I think the thing that's been good is it’s a stroke and neuro project, not just one or the other …. And I think this is really helped stroke and neuro therapy staff to get to know each other better, work together and have that kind of cross pathway work for our patients. *S11:* we probably worked a little bit more collaboratively than I would have done if I was just seeing someone on my own.
**Readiness for implementation**	Staff	NROL needs leadership commitment from clinicians, management and clinical academics	S01: I mean the people behind it. You've got the therapist themselves, which do an absolutely wonderful job. They’re amazing at it. But you've got people like (clinical academics), who worked tirelessly behind the scenes to try and pull everything together and make sure everything runs smoothly as possible. S07: I know particularly my operational lead, and service lead as well, has been really positive and really pushing NROL. She really sees the benefit, so we have been supported from that perspective.
		Clinical academic partnership and funding from the ‘SameYou’ charity a key enabler for implementing NROL	S03: because we already have this partnership between the UCLan and ELHT …. but without that it just wouldn't have happened. I have a feeling that a lot of it would have just gone on the ‘too difficult’ pile. S14: I think it's been well supported once we could evidence the impact of it… I think we wouldn't have got it off the floor if we didn't have the charity funding. I don't think we would have got it anywhere near to what it is.

### Mixed methods integration

**Fig. 3:**
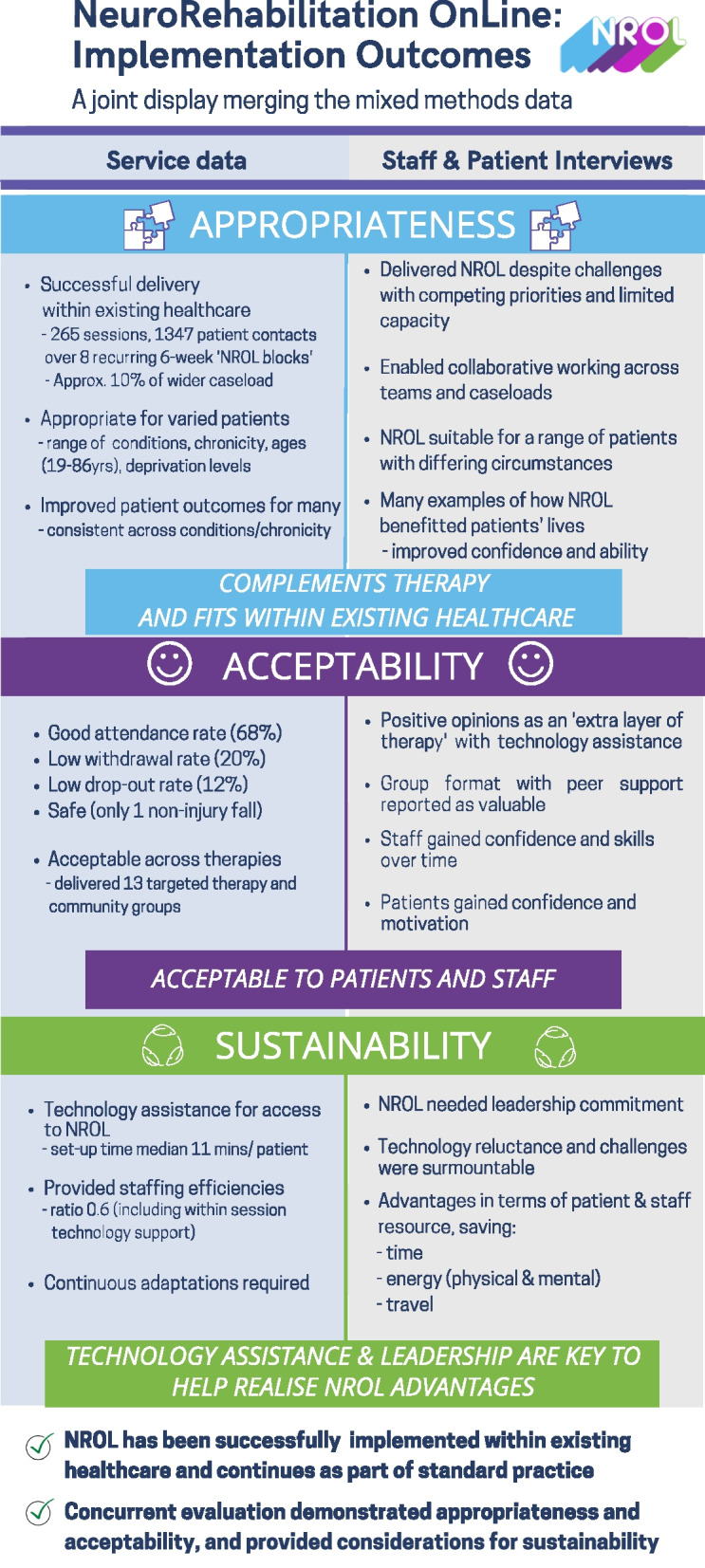
Joint display merging mixed methods data

A joint display (Fig. 3) integrates the analyses to explore the implementation outcomes of appropriateness, acceptability, and sustainability.

## Discussion

The current project provides an exemplar of concurrent implementation and evaluation of a telerehabilitation approach. Pragmatic implementation of the group-based telerehabilitation, NROL, was possible, well-attended, and demonstrated appropriateness to complement existing therapy for patients receiving community-based neurological rehabilitation. It was accepted as a new way of working across rehabilitation disciplines as an ‘extra layer of therapy’. NROL had perceived advantages in terms of patient and staff resource (e.g. saving time, energy and travel). NROL provided staffing efficiencies (ratio 0.6) compared to one-to-one delivery. Technology difficulties and reluctance were surmountable with dedicated technology assistance. Leadership commitment was considered key to enable the efforts needed for implementation and sustained use. NROL continues as part of standard practice. Evaluation was undertaken in a timely way to consider fit within existing healthcare from the outset.

NROL was compatible with usual service provision. It was appropriate for mixed demographics, including patients with a variety of neurological diagnoses (sudden, intermittent and progressive conditions) and chronicity. This breadth reflects the reality of neurological rehabilitation services but also creates challenges for group content. However, findings indicate delivering groups to patients with varying conditions and chronicity was suitable given there were no associations between these characteristics and direction of change in patient outcome scores. Additionally, benefits were identified by staff including enabling collaborative working across teams and caseloads. Relative to the wider caseload NROL patients were likely younger. A factor could be gate-keeping from therapists, as there was recognition NROL is not suitable for all patients which inherently requires decision-making. NROL was provided to patients from diverse socioeconomic backgrounds, and most lived with a family member or carer. Of note, we did not have any care home residents participating in NROL despite a likely need. Equity considerations warrant further investigation to ensure that embracing technology does not increase inequalities or create new ones.

Patient outcomes indicated improvements for many alongside emotive examples of how NROL benefitted patients’ lives. Echoing the results of Beare and colleagues [24], we found statistical improvement in self-efficacy. Additionally, we found that patients rating of their ‘ability to perform functional activities that were important to them’ increased by a similar magnitude to those reported following multidisciplinary in-person home-based rehabilitation with older adults [40]. Even with the relatively short time-period of the NROL programme, and the wider context of the on-going pandemic, health related quality of life also increased. Despite the caveat that group level improvements did not exceed referenced minimal clinically important differences, many individuals had scores that remained stable or improved for their self-efficacy, quality of life and activity performance. Several of these improvements were to a potentially clinically meaningful degree, which is notable given the inclusion of patients with deteriorating conditions. Overall patient outcome data align with the interview data which imply patients felt NROL helped from a physical, and mental health perspective and supported them to gain confidence and motivation outside of NROL. However, it was emphasised by both patients and staff that NROL must sit alongside in-person therapy, rather than replacing it. It is known that current therapy dose falls short of what is required in clinical guidelines and there is a recognised need to optimise opportunity to rehabilitation [51–53]. We demonstrated that telerehabilitation delivery methods could offer a compatible way to increase therapy and help meet guideline targets, but it should supplement rather than substitute therapy provision [12].

Telerehabilitation provided the opportunity to develop a new skill set for staff, with technology support being given to staff as well as to patients to enable their successful use of the online platform. Staff confidence with technology use increased over time. There has been suggestion that specific training is required for staff to deliver telerehabilitation [23, 54]. We found experiential training with technology support to be sufficient, which confers with Lawford and colleagues that first-hand experience of this method of delivery is key [55]. The staff:patient ratios reported reflect not only the staff delivery and technology assistance during sessions but also within-session staff training. The data captures a relatively early snapshot of delivering NROL, with the numbers of qualified staff per patient reducing over time. Telerehabilitation enables extra staff to attend as a way of sharing learning with newer team members and students, and potentially shadowing a virtual session is easier than being an extra person in an in-person session. This is important for sustainability as it grows skills that will increasingly form part of prospective service delivery and as future patients are more comfortable with technology.

As a concept, telerehabilitation has many advantages which were conveyed in our evaluation in terms of making the best use of the limited resources of both patients and staff. Telerehabilitation aligns with many current policies and priorities [1, 2, 56]. Strategic drivers include improved patient choice, managing workforce challenges and effective use of resources, exploiting the potential of digital technologies, and reducing carbon emissions. These drivers will continue to be relevant long after COVID-19 has receded thus to enable advantages to come to fruition, there needs to be investment. The implementation of NROL did take resources, time and effort which required buy-in and commitment from leaders (clinicians, management and clinical academics). These resources needed for implementation are not unexpected [6, 57] but current healthcare systems are often not set-up to facilitate this upfront effort. Going beyond initial implementation, the sustainability of telerehabilitation approaches are likely to need some service redesign [58]. For example, we identified that incorporating technology support roles within staffing allocations will be required. It can be a challenge to enable long-term sustainability as this requires buy-in from the stakeholders who have authority for system change (commissioners and managers). Arguably, influencing key stakeholders using data to demonstrate need and benefits of telerehabilitation enhances the likelihood of service redesign to embrace this new way of working.

The impact of COVID-19 meant that we could not wait for stronger evidence of effectiveness before implementing telerehabilitation [13]. This was not an effectiveness trial, rather it explored an alternative delivery method for neurological rehabilitation to help inform future practice. For NROL, the content is similar to in-person rehabilitation and existing effectiveness evidence shows telerehabilitation is not inferior to conventional delivery [16, 59]. As suggested in guidance on complex intervention research, the implementation and evaluation phases are not always sequential [60] and in this case are concurrent. We asked broad questions about NROL, assessing its value from multiple perspectives. We adapted NROL to fit within a healthcare system and evaluated this telerehabilitation approach in a rapidly changing context. The use of traditional randomised controlled trials was not fit for this purpose. A pragmatic, pluralist and timely approach to evaluation is required [60], with the use of implementation frameworks advocated [13]. This has many parallels with action research. We used the Consolidated Framework for Implementation Research [44] in our qualitative data collection and analysis and selected Proctor’s implementation outcomes to structure our evaluation findings. A challenge was that there is no clear consensus on how to define or use Proctor’s implementation outcomes. For example, what is deemed as acceptable and according to who? Historically, quantitative methods e.g. [61] or qualitative methods have been used [62] to deem what is acceptable. We used a mixed methods approach to ensure a comprehensive understanding of appropriateness, acceptability and sustainability from multiple perspectives, with triangulation from different stakeholders and methods. Going forwards, more discussion of how implementation outcomes should be defined and evaluated will help progress the field in generating broader evidence that is timely and fit for purpose.

### Limitations

This was a pragmatic and constantly changing implementation and evaluation of a telerehabilitation programme in a changing context. Data capture was difficult, both from being able to extract from electronic systems within healthcare and consistent outcome measure use. Specific examples include it was not possible to extract comprehensive data on ethnicity, and it was also difficult to capture what was not done (e.g. who isn’t referred). Effort was made to capture data from outcome measures in current use (to improve resource efficiency and assist long-sustainability) but support was needed to ensure adequate data collection. NROL was delivered to patients with varied diagnoses, which has challenges with validity of outcome measures for different populations. With respect to the interviews, there is potential positive and social desirability bias, and the timing of the interviews was relatively early in the implementation so opinions may have changed over time. Although the conclusions regarding NROL advantages were primarily drawn from this interview data, a previous study has demonstrated quantitative service and travel efficiencies [4]. However, it is acknowledged that further evaluation combining qualitative and quantitative data would be beneficial, as would incorporating a cost analysis.

## Conclusion

Pragmatic implementation of group-based telerehabilitation was possible within an existing healthcare system. Providing NROL as an adjunct to neurological rehabilitation has compelling advantages of in terms of leveraging patient and staff resource, and staffing efficiencies, supporting the continued use of this telerehabilitation approach. Additionally, NROL was seen as an opportunity to develop new professional skills and evolve existing roles. This project provides an exemplar of how evaluation can be run concurrently with implementation. A data driven rather than anecdotal approach is needed to harness opportunities afforded from the COVID-19 pandemic, rather than reverting to ‘normal’. Continuous evaluation is required to understand more, including on equity, and future delivery should explore how telerehabilitation can be upscaled and fit within a changing context.

## Supplementary Information

**Additional file 1.** NROL Staff Interview Guide.

**Additional file 2.** Interview analysis using CFIR domains.

## Data Availability

The datasets used and/or analysed during the current study are available from the corresponding author on reasonable request.

## Abbreviations

CFIR: Consolidated Framework for Implementation Research
ELHT: East Lancashire Hospitals National Health Service Trust
NROL: NeuroRehabilitation OnLine
MCID: Minimal Clinically Important Difference
MS: Multiple Sclerosis
PSFS: Patient Specific Functional Scale
SSEQ: Stroke Self-efficacy Questionnaire
TIDieR: Template for Intervention Description and Replication
