# Supplementary material for: Implementation of neurological group-based telerehabilitation within existing healthcare during the COVID-19 pandemic: a mixed methods evaluation
Source: BMC Health Serv Res. 2023 Jun 21;23:671. doi: 10.1186/s12913-023-09635-w (PMC10283243; doi:10.1186/s12913-023-09635-w)
Supplement: Supplementary file 1 — Additional file 1. NROL Staff Interview Guide. [file 12913_2023_9635_MOESM1_ESM.docx]

| **INTRODUCTORY QUESTIONS** | | |
| --- | --- | --- |
| - Can you tell me about your experience in neurological rehabilitation? - What is your current role? | | |
| **NeuroRehabilitation OnLine @ ELHT (called NROL throughout)** | | |
| - Can you describe in your own words what NROL is? - How important was the technological support for enabling NROL? - Did you have any issues with having the right devices/ connectivity in the hospital? | | |
| **CHARACTERISTICS OF INDIVIDUAL** | | |
| - What where your expectations and motivations for delivering NROL? - How confident were you about the prospect of being able to deliver NROL? - What were your experiences of technology/ remote rehabilitation before delivering NROL? | | |
| **COHERENCE** | | |
| **Dimensions** | **Questions** | |
| **Differentiation**  *(Is NROL perceived to be different from traditional ways of working?)* | Was the NROL programme the sort of thing you expect to be part of rehabilitation? | How does it differ from the ‘usual’ ways of working? |
| **Communal Specification**  *(Does everybody understand NROL?)* | Before starting NROL, did you understand what the NROL programme was? What about the rest of the team? | Did you think the purpose of NROL was clearly explained? |
| **Individual Specification**  *(Does everybody understand what they have to do when using NROL?)* |  | Did you understand what you had to do in the NROL sessions? How should we explain it to people in the future? |
| **Internalisation**  *(Does everybody think it is worth the effort?)* | Did those around you (carers, other family, other patients or therapists) think NROL was worthwhile? | Did you think NROL was worth the effort? |

| **COGNITIVE PARTICIPATION** | | | |
| --- | --- | --- | --- |
| **Dimensions** | **Questions** | | |
| **Initiation**  *(Are there key individuals that advocate for NROL?)* | Were there key people that helped in getting NROL off the ground? | | What strategies helped to get NROL started? |
| **Enrolment**  *(Have people “bought into” NROL?)* | Did being involved in NROL influence anyone around you? | |  |
| **Legitimation**  *(Are the right people doing the right tasks?)* | Did anything get in the way of doing NROL? | | Have you had to make any changes to get NROL working well? |
| **Activation**  *(Is everybody ready to make a plan?)* |  | | Did doing the NROL programme affect how your day/ week was organised? |
| **COLLECTIVE ACTION** | | | |
| **Dimensions** | **Questions** | | |
| **Interactional Workability**  *(Is the work involved in delivering NROL appropriately allocated?)* | Did you feel there was the right balance of groups from physios/OTs/ SLTs/ psychology? | Was there the right level of involvement from different staff members? | |
| **Relational Integration**  *(Do staff and patients trust each other’s work and expertise in using the NROL?)* | Are you confident that staff have the expertise to provide NROL programmes? | Did you feel safe delivering rehabilitation remotely? | |
| **Skill Set Workability**  *(Can people perform the tasks that are being asked of them?)* | Did people have the right skills and knowledge needed to deliver the NROL programmes? | Has there been any training provided? Any gaps? | |
| **Contextual Integration**  *(Is NROL adequately supported by the host organisation?)* | Was there sufficient support from the NHS/ rehab teams to provide NROL? | Is there anything in particular that supported the provision of NROL? | |
| **REFLEXIVE MONITORING** | | | |
| **Dimensions** | **Questions** | | |
| **Systematizing**  *(Is implementing NROL worthwhile?)* | Do you think the NROL programme was helpful? Prompts: from a physical, cognitive and/ or social perspective | How did you gauge if the NROL programme was working or not? | |
| **Communal Appraisal**  *(Are people finding implementing NROL a worthwhile venture?)* | Do you think other staff and patients think NROL is worthwhile? |  | |
| **Individual Appraisal**  *(Do individuals evaluate the new practice as worthwhile?)* | Do you think it is worth continuing to use NROL as part of rehabilitation? | Have you noticed any changes in your practice in general? (related to NROL) | |
| **Reconfiguration**  *(Do people modify their practice in response to evaluations made?)* | Would you make any changes to NROL based on your experiences? |  | |
