# Supplementary material for: Implementation of neurological group-based telerehabilitation within existing healthcare during the COVID-19 pandemic: a mixed methods evaluation
Source: BMC Health Serv Res. 2023 Jun 21;23:671. doi: 10.1186/s12913-023-09635-w (PMC10283243; doi:10.1186/s12913-023-09635-w)
Supplement: Supplementary file 2 — Additional file 2. Interview analysis using CFIR domains. [file 12913_2023_9635_MOESM2_ESM.docx]

| **Characteristics of intervention** | | | | |
| --- | --- | --- | --- | --- |
| **Construct** | **Staff** | **Quotes** | **Patients** | **Quotes** |
| Complexity | Lots of elements, an effort to get going  A new way of working for staff and patients | S04: it was new, we had to develop our group… so that was a lot of work initially… I was a bit unsure, a bit nervous about how patients were going to interact  S14: it would help with waiting times and getting a larger volume patients seen at the same time. Obviously, the added influence to that is the time taken to then set up the program and set up the sessions does take away from some of clinical time we have. So it’s kind of weighing that up as well.  S02: what we don’t know is the overload of both doing the intervention and doing it online…  S01: A lot of it was just trying to problem solve with many different devices trying to access the same platform… all reacting in different ways … in all cases there’s always been work arounds  S01: we’re learning all the time, and things may change, the way it operates may change  S04: as therapists, we have to work with it, just the ins and outs, and top tips of using Teams | Importance of having support - At home   - Technology support   Need the correct set-up  Technology reliability and consistency | P01: what I noticed is that when we were on the zooms, nearly everybody else, I’d say 80% of people, needed somebody there to physically get them on the zoom  The only thing that would make it difficult for people is the technology, lack of familiarity with it and physically getting on…. And that’s why you need a person like (tech support) and, you have to clone him.  P03: you do need to have a kind of room where you’ve got the facilities to be able to support yourselves on something, or a wall’s got to be close to you  P14: it was difficult in the fact they want to see your leg movement… I couldn’t get it (laptop) far enough away to see the whole of me  P06: but then you get the idiosyncrasies of Microsoft…. Sometimes you don’t get what you expected |
| Design Quality and Packaging | Importance of preparation of group content- varied by group  Group names and content has evolved | S04 (OT): ‘cause you can’t just really wing six weeks worth of therapy… we did have to do a lot of prepping  S11 (SLT): we had a brief structure, but we didn’t really know what we going to do in week 5 until just before week 5… we didn’t know what level to gauge it at until we met the patients…  S06: They’ve got fancy names now. And I think café NROL is a fantastic one, because you know that allows the social… they are still socially deprived with what they can access  S09: I don’t think by any stretch it is a finished product, but I think it works quite well, and it fits in with organisation and planning  S15: I think we set up the groups that we think we can run and then find patients to fit them…. we’re not quite yet able to go, we’ve got this bunch of patients, let’s make a group around them… we’re not quite nimble enough | Importance of having experienced therapists  Presentation of NROL | P01: you need more than ever to have expertise, because you can’t pick up the subtle nuances… I felt very safe because you had the trust in them and you knew they had the experience  P06: communication is the key…. The teeing up of the session about expectations of you, the client, the therapist, and the project team. The contracting stage is crucial.  P13: I didn’t think I was going into it blind. I think the pace of it is quite ok. If it would be more structured… it would probably like put me off |
| Intervention source | Informed by external source (UCL), but internally developed/adapted as a good idea, solution to Covid | S02: met with the people delivering the therapy down at UCL. So they’ve learned from them, they’ve built on what they shared… They (UCL) were very different in terms of service model.  S15: I knew the model from what UCL had done..so I had a vague sense that it would be something like that… but I had to do NROL alongside all the other stuff.. so I knew it was going to have to look a bit different | Minimal awareness | P05: I didn’t fully understand the connection between NROL, UCLan and the others |
| Relative Advantage | Virtual offers advantages in terms of time/ transport/ less fatigue for patients  Peer support key- more important than anticipated  Advantage: improved teaching/ education elements   - Encouraged joint working (across disciplines and pathways) | S02: I think with NROL we definitely did bring more group therapy than we would normally do face to face. So we have groups running now that would not be the norm.  S02: if you're gonna run face-to-face groups where you've got a whole host of problems, not least you need to find premises, you need to deal with the transport issues, you need to deal with the care issues, you need to deal with the practicalities of toileting and wheelchairs and drinks, and all the rest of it, and so actually there's a reluctance sometimes to develop groups face to face, because of all the complexities. But this is an easy way of doing it.  S03: because in the long run it's you know it is saving time, so even if for example it takes a bit of time to get somebody tech savvy, ..then you don't have to visit for five weeks. That weigh-up of time seems very reasonable  S09: within the hour or the hour and a half that I've kind of got booked out to run the group, I can see four times as many patients  S06: it's just that accessibility of it that you, you know, you know you've got one hour, but you’re probably seeing six different people. It's just looking at it, it's just a different way of looking at things  S11: I know from a community side of things, we spend so much time travelling. And it just took me 40 minutes to get from my last visit to here ... And then in that time, we could have done a group. And so I think that that's really effective.  S13: in terms of, I suppose, efficiency to some extent, in that we're not having to travel to five different patients. We can see them all in one go. So it's good from that respect. So I suppose from a, if I was talking to a commissioner, I'd be pitching NROL in that respect in that you can see more than more than one patient in one go, you know, having to travel to you're not claiming on expenses, I suppose from an eco friendly point of view, you're cutting down on your carbon emissions as well.  S15: one of the sort of fringe benefits of pushing tech a lot more in the pandemic. So like we used to do some face to face groups pre Covid and I never ran any because I thought just getting people to a base would be so difficult. You end up with such a tiny subset of people that you actually work with, it becomes more effort than is actually justified. So I think one of the things that's really good about having the online stuff is it does give people more options. It means that you can turn up without having to go anywhere. It means that you can deliver the group session without having to also factor in an hour plus for transport and setting up and making brews and all that sort of thing. So I do think that bits really good.  S03: I think even as things get back to normal we still have a lot of patients who are isolated anyway because they can't drive anymore, they just see the family and the carers that come in. And you know that contact with other people through a group session is great.  S03: So if that peer support can give them the feeling of not being the only one…the sharing of the difficulties. If that can help with their wellbeing side of it, then they're gonna feel more motivated to do the therapy to get better. If they're not motivated to do the therapy, then you know as much as we can try again and speak to them about it, we can't physically make them do it, so I think that wellbeing side of it, it really helps toward it as well. It's massive  S03: so they get to know each other and then they get to learn from each other. I'm not the only one that can't keep track of a conversation or. I lose lots of what's told me, it helps them to know that they're not on their own, and other people feel like that or other people actually get better. You’ve just got to take more time, learn to be patient with yourself so that the patients learn from the peers in this group forum which is really nice. Which you wouldn't get you would get from a physical group if you were able, or one-to-one sessions.  S07: the patients are hugely enjoying NROL from a kind of a social point of view, obviously as well as the therapeutic benefits that they get in, and I think particularly Cafe NROL and the lifestyle discussions, they find it really useful to speak to people in similar situations  S08: with cognitive rehab, there would be an element of education, but it would be on a one-to-one basis but it would be very much talking to the patient in their own home and giving them information leaflets to talk to them about. But I think the difference that we found with running the groups is that patients are sharing their experiences with each other and I think they’ve actually taken on board the education better because they’re hearing it from each other not just from a therapist who hasn’t experienced having a brain injury.  S08: I guess some of the feedback we've had is they understand why therapists are getting them to do certain tasks at home and how that helps with neuroplasticity and brain recovery. And even though I know patients get told that in one-to-one sessions there's lots of things that we repeated, for some reason, doing it in a group together seems to have, I don't know, I think they seemed to understand it better, took it on board better because it's not just the therapist telling them  S06: I think NROL has helped people be more self-sufficient. So you know, sometimes, and we do say this, we plan the patients appointment, we go to their house. You know, there's not an awful lot they have to do to make an effort, and so the NROL has you know, put the responsibility in some ways back onto them, to be ready for the session, erm organize the link etc etc, which has then made, probably made them feel a bit more of an achievement when they've done it.  S15: I think there is something about people actively remembering, proactively remembering to show up. It's all that prospective memory stuff and planning and actually caring enough to put memory strategies into place to show up. And they're happy enough to show up, but they sort of haven't really invested in making sure they show up themselves. And I think a lot of our patients, they used to just knock on the door and there’s the therapist. So it's a really different way of being. I think we've underestimated how different that is and how much nudging and support people need sometimes to do that.  S02: is that giving us an opportunity to kind of connect some professions with patients that wouldn't do normally…You know, neurologists don't normally come to group therapy sessions. But asking them to deliver something online like this suddenly is easy for them to do, and that strikes me as being a great opportunity for patients that they wouldn't normally get, and an easy way to bring other disciplines in.  S11: we probably worked a little bit more collaboratively than I would have done if I was just seeing someone on my own. So we did some inferencing and I've never even really come across that much and we did a whole section on that, so it was very little speech and language focused. Which was good for my learning …. But it's not something that would’ve normally touched on.  DISADVANTAGE  S15: I think the bit that I find a bit tricky is it sort of just being able to get a sense of kind of like if you get people face to face, you sort of take the temperature of the room, you can work out where people's heads are at. You can tell, I think, a lot more about just generally how somebody is by just the manner they've got. And I think you do lose a bit of that sometimes via Teams.  S15: I suppose the other bit that I talk about, but I'm not sure how easy it is to manage, is thinking about things like confidentiality and group boundaries and things like that. And I do think that perhaps that makes me veer towards more of a light touch, psycho educational type of group that's a little bit more like delivering training and discussion rather than if you've got people all sat around in the  same room face to face. You kind of get a lot more into the nitty gritty of things. But I don't know who's listening in, to some of those confessions. You hope that people are on their own, but some people are family members because they can't manage it themselves.  S17: So you've got no hands on ability, so you've got no ability to correct somebody's… from a physio point of view, no ability to be able to correct their technique and exercise | Virtual offers advantages in terms of time/ transport/ less fatigue for patients  Patients reported feeling valued/ a role themselves in supporting others  - Advantage: improved technology skills | P01: I think it’s beneficial to the Trust. I think they're missing a trick if they don't roll it out more, because you know it must save them money to treat all these patients in one go.  P03: 'cause one of the things I quite quickly realized… was it was actually really good to see how I was comparing against the other people that were taking part. You don't get that from a physio one to one, but you do get it if you're doing it with a group on video 'cause you can actually see where some people are doing better than you. And, where they're doing not so good.  So you don't even get that kind of insight when you are in a one to one situation at a hospital, because you’re always seen as an individual, and not until i did the NROL exercises I didn't really realize just how actually important it is to maybe do things as a group, with people who were all suffering similar kind of things.  you know that somebody else is going through the same kind of things that you are. Which normally you don't feel that, you know ‘I'm the only person in the world that's ever had this problem’. No I’m not, obviously.  I was shocked that they could see just about everything I was doing. So yeah that, after I think the second exercise session I thought, now this is almost as good as having the physio in the room and in some ways it's slightly better because you've got two physios in the room. I think that was quite valuable to be honest, having two of them.  P06: from a client perspective it's less difficult to get in and you don't have to drive…. I personally didn't encounter any difficulties by doing it online and I found it very beneficial and the follow up material that we've had for the physio particularly has been excellent.  I've always extolled the benefits of doing things within a group. You build a bit of a camaraderie, you get shared learning and experiences.  it's a good model to use. So there's the therapeutic effect... and we had fun learning and there was still a tangibility at the end of it  you’re not tired when you arrive…I used to joke it's a full time job being ill because. I use patient transport so it can be half a day to a day …Well, this is short sharp burst. It's easy, it's manageable, and you can do it. It's done and you can integrate it then into your life. If it's a speaking thing or it's a physical thing, you can recuperate faster and you can do some more.  this for me is a low maintenance way to deal with therapy full stop, but a really good way to deal with people who've got chronic ailments where it's a long term thing and you've got a really cost effective model to stop them chomping away at your hospital resources.  P13: when I go to appointments I always end up relapsing and then I'm in bed for like a week. So if I was doing this every week, I would I would just, you know, I'd go to the session and then be ill for the week so I'd have to decide whether or not it was worth it. But obviously if it is done remotely or online it's so much easier, because the physical element of it is, you know you don't have to think about it then.  P01: some of the people that were with the relatives when they were doing the exercise., you know, I could tell they were really appreciative....And I suppose when they're listening, they can reinforce the treatments that they’re getting at home. Whereas when you’re sometimes in hospital, you might just have, like, my husband, usually drops me off and then you go into the physio, perhaps a lot people, they just go in on their own and the relative might wait outside, whereas they might not know what the physios want them to do. |
| Adaptability | Continuous refinement/ adapting &  problem-solving  Phased implementation- more groups over time  Staffing make-up changing over time | S01: we've always found work arounds to get everybody that needs to be on these therapy groups to be on  S01: we're learning all the time, and things may change, the way it operates may change, and hopefully we'll get the current system to work in our favour …  S02: I think we've been opportunistic with it. We don't really know what suites of therapy groups we’re going to be delivering…if this is really gonna work, then we may have a core suite of groups, but actually our suite of interventions should expand and change overtime.  S01: I think as our experience grows and as the proof of its benefits become known, I think more therapy units will join in and possibly adopt the system as well.  S03: And we can always be doing stuff alongside NROL….you know, NROL might just mean instead of doing two visits a week that you do in one visit a week to reinforce what they've just learned in NROL and then take it a bit further because they need that extra kind of input and knowledge and support. It adds a lot of flexibility I think  S14: I think the vision going forward, would it be that they could access NROL twice a year or once a year just as a top up, rather than actually having to have face to face therapy if they didn't need it, just to keep them motivated. And obviously building this community from NROL, the patients then make something themselves, and something they can kind of continue with the sort of self-management, whatever it is, just to meet up - a coffee morning or whatever it is, they actually meet up and go to the gym or this that and the other. It depends really. And I think. And I think that's the main aim is, is - can we build a community of patients that can almost look after themselves with a little bit of input from us when they need to.  S04: I think from a content point of view, we will be guided by patient feedback and the more patients we're seeing come through, we might get different feedback from a different cohort of people  S13: another example of the way NROL’s evolved, is that I think originally we thought it was just going to be purely online, whereas I suppose in some cases it needs to be a bit of a hybrid model, a combination of online therapy sessions and also physical kind of hands-on therapy sessions as well.  S02: we've started with the initial physical ones. We've got cognition and communication both now on board, we've got psychology on board. We've got something about life after brain injury on there. So I think the broad spectrum’s there now in all of them. But I think there are so many different ramifications of what kind of groups you could do. I think that should constantly change. So I don't think we should be bound by these four group types, and we almost always deliver those four group types. I think we must constantly look at what the biggest demand for interventions are that can be delivered remotely, and then what opportunities come when you have either clusters of patients or new interventions. So I don't think that should be ever be fixed  S03: So it's good that it's not like set in stone that we're looking at what's actually coming through rather than trying to drum up business for something and putting people on it that potentially aren't correct for it.  S09: And I think there's room for development. I don't think we've exhausted all options (re: groups) yet  S15: We need to make it work for us rather than us working for NROL. But I think it's taken quite a bit of time for that. I think people are still a bit unclear about exactly what all the different groups do. But I think people are more willing to just sort of. Give it a go, rather than thinking they've got to perfectly understand it and perfectly refer someone to the perfect group. So I think we're getting there, but it's a slow process  S14: got to be very wary that we've got to be flexible, … The group should meet the needs of the patients, not the patients meet the needs of the groups. We should constantly review what the groups are offering as to the patients that are referred, so making sure that it kind of tailors to their needs, not ours  S13: With the capacity we have and the number of referrals and patients we have it wouldn't be physically possible to keep patients on a running program like that at the levels we've got for six months. So I think six weeks works well for us, but then is the model going forward’s that patients can re-attend after X amount of time, or when they need certain sessions, or is it they kind of do a step by step, so some have been doing sort of one group for one block and then as they've progressed with that, they move on to a different session. So I think one is speech and language they did speech and language, specific group, and then they did a book club the next block, so kind of a bit of a progression to the point where then they could self-manage. But I think the longevity is there cause the patients are going to be there, we will continually get patients referred.  S03: so far the majority of it is being run by, qualified therapists, which is fine because it's been a new thing, but when you look at some of those therapists, a lot of them are band sixes and band sevens, which to get things off the ground and to make sure it's running well is, you know I can understand why … Once things are a bit more established, I think we need to play about with the experience levels or the makeup of the two people together that are running the groups and utilize assistants a bit more potentially.  S07: Yes, there needs to be two members of staff, from a safety point of view, but we are just starting to consider the use of our assistant practitioners a little bit more. And to be that second person. And I think from looking across the workforce, I think that that would be more appropriate going forward. | Able to tailor  - difficulty of therapy  - carer involvement (or not)  Responsive to feedback from patients  Ideally would like more/ suggested ‘add-ons’ | P01: it was good as well because then …he’d see some things I was struggling on ‘well you could perhaps just do it from sat down’, so they could modify and I think that's the benefit as well. From the zoom you know you could see everybody from their point of view, and he could say well if you just do this from this position you just do that from that position. So it was good.  P01: You know, some people have relatives with them. You know what I mean. So they got a bit of instruction as well, I suppose, because they could make sure that they were supporting that person there.  P06: We got the feeling, it's bespoke really. And to be fair a lot of this has felt bespoke which is nice and rare.  P06: I must admit I did suggest some elements, as did other people on the event about having videos that we can play and repeat. You know, it's great when you've got the physio guy on, but it's also fabulous to watch him do it. Here is tips and pointers and then do it yourself, so that's good too.  P10: This might just be me. In terms of using things like WhatsApp or something like that …you know that was sent out the group, you know everybody even to do. This is your task for today to perform this ten times or whatever, and that kind of thing might have been great …Yeah, that's just one extra thing.  P09: I would feel like an extra two maybe three sessions would be good**…** I said make it a bit more longer, …would be of benefit |

| **Characteristics of individuals** | | | | |
| --- | --- | --- | --- | --- |
| **Construct** | **Staff** | **Quotes** | **Patients** | **Quotes** |
| Knowledge and Beliefs | conflict with quality of movement v quantity- fit better with some people’s belief system than others   - Concern around increasing not replacing   What is core business v nice to have?  Peer support | S11: Provided a lot of opportunities for practice, and to learn new skills and even remind them of some of what they've already gone over and on OT sessions or whatever, just drilling that in a little bit. A lot of therapy is repetition. And so it's really good just to sort of hammer home what they need to learn.  S03: quite a few of the patients are quite fearful about going out into the big wide world again, …. Because they’ve been so isolated, so I think it has been a big part of it, but not detrimental to the therapy. And, you know, probably very helpful. Probably just as therapeutic as doing those physio exercises or that speech and language exercise because of the mental wellbeing. Yeah, because they’ve not been getting the contact with anybody. And a lot of them are fed off each other’s - they've been sharing like best tips and or ‘I’ve been passing the time by doing this game on such an app’ and or ‘I've been writing letters’, or you know, or just sharing stuff like that. It's been really good.:  S03: there's a couple of patients that I’d put through onto the cognitive group who only have I suppose, in the scheme of what we see, quite mild cognitive difficulties, but to them they seemed massive because it it was a change for them, so putting them, so, giving them the knowledge of the group, but also that seeing that It could be worse kind of situation has made them really crack on with their lives and think, ‘right, OK, let's stop feeling sorry for myself. Let's get on with it’. I've had some patients who were quite a long way down the line from the brain injury like several years who have said ‘oh if id have done this eighteen months ago I wouldn’t have thought that I had any problems because I were too early on’ or whatever. Whereas the’re now seeing having done it now they're looking back and thinking, ‘right, well, I definitely had that problem and I definitely had that problem’. And then you've got people who had quite acute in the brain injury who it's helping give that knowledge to, give that purpose to. To do an exercise at a particular time on a particular day and then do something else the day after and something else the day after and just give them the strategies, the memory strategies from the OT Group, and the speech and language as well, just you know. I think the fact that at the minute from a speech and language POV, if people are stuck at home so much, you speak ito maybe your family and they're getting used to how you're speaking, and but then potentially when you see other people that you've not seen for a long time or never seen before like in the supermarket or whatever, you’ve not then got those skills to be able to get across what you're trying to say properly. So the fact that they've been able to, in a group setting, get some skills together, get some techniques and practice it, I think has given some patients a lot of confidence to be able to go out and do some things again  I think that there was to an extent an expectation of it, because that's why they kind of put the cafe NROL roll side of it together as a peer support kind of thing, but I think for the people that we've had so far, it's worked far better than what we imagined it would do.  S05: Definitely, you can see by the end of the block them coming out of themselves more and you can see the strategies that they've built on and they've taken feedback about what they've done to help themselves … an example was given yesterday. He's relating how difficult his journeys are and obstacles that can come up then it that's showing that he started to reflect. That's just from the second session he's already reflected on his days and how well he problem solves. And you know you can see like the minds are starting to tick over already and really start to analyze what they do and what other strategies they could put in place, and also from by the end of the two previous blocks. Anyway, by the end of it the final session there, they were all chatting away and smiling and even talking between themselves. Like I said at the start that start the session when it's that informal chatter, they'll be talking to each other and one lady actually said that she had found the confidence to take up piano again. She felt that she was at a point, and there's definitely been stories and a lot’s come out of it as far as we know.  S09: particularly starting an upper limb and just wanting healthy discussions and debates about how to grade it, where to start, which patients to choose, erm, whether things were lost in translation I'm not sure, but I know there was a lot of resistance about who does it? How long is it going to take? Oh we've got to make sure we're offering X , Y and Z to the patients as well as NROL, and that NROL is not a substitute, that's always been pushed. That we are still seeing patients face to face. I think that's been the primary resistance.  S15: Because you run a group and people are really enjoying that. So that's lovely. But I can't just run a group that you enjoy. Because then I'm not doing something else that someone really needs. So you've got to enjoy it and need it, and to benefit from it as well.  S15: I think some people were worried that we were going to be pushed into sort of delivering this because it was prestige thing and it would look impressive.  S07: I think as a therapist and as a physiotherapist, particularly working in stroke and neuro we’re very hands on, and so we're always trying to work for a normal movement pattern. We want to encourage people to work in the right way and develop strength in the correct muscles. And I think when you're delivering sessions virtually - yes you can verbally prompt and yes you can, you can try and correct that as much as possible - however, for your lower level patients they do very much need kind of hands on guidance, hands on therapy to promote the effectiveness of the treatment. And I don't feel as a therapist, unfortunately, that delivering that virtually would give the same quality of therapy potentially to giving something face to face. And again, I alluded to it before, from a balance perspective - yes, you can challenge balancing. Yes, there are ways around it. You can have family members and carers supporting, and therapists doing their best to make sure they're adapting and progressing. But when you're in a balance group, if you've got a therapist there and give that closer supervision, you are able to just push them that little bit more and just get that extra little bit out of them. | Concern around increasing not replacing  Positive experience- felt it helped  Peer support  Varied reasons for taking part | P03: But I was just concerned …because one I didn't want to give up the home visits, because the physios all come to me, and that was like super valuable  P01: I prefer the hands-on physio really… but the next best thing is this  P02: Yeah, more confident. I can walk into a shop now. Talk to someone and actually tell somebody what I want as opposed to just walking out.  P03: One was me ending up after the exercise session in a complete sweat and absolutely knackered [Laughter]. Which is always a good sign. And the other bit I kind of said before - they can see what you're doing and what the issues are with what you're doing, and they can give you advice on how to correct. I didn't think they could do that through a video link, but they can, they absolutely can.  P03: Bonding and learning. But also we make slow progress. It's the nature of what's happened to us, so our progress takes a lot of time to develop, and sometimes you don't realize that you are making progress yourself. So, if we allow more time, people get to know you a little bit better and they can give you feedback on whether you're doing better or not. And that's really important to get somebody to say to you ‘that’s really improved since two weeks ago or three weeks ago’. That that's a massive boost.  P07: And that was brilliant because you just get to talk to each other about you really.  … And they've been so friendly and so helpful. And It's helped me move on. It really has. Yeah, especially during covid. I live on my own and it's just amazing and I've learned so many different things.  P01: To me NROL is an extra addition to a person's normal therapy and it just provides an additional layer of therapy to a person where for instance, in lock down It's been that ability to get therapy where therapy wouldn't be available.  P05: it meant so much. Because between appointments, you tend to feel abandoned nobody is listening, nobody is bothered by what problems you have, and this a way of discussing things, you know, in the interim. So for me, it's just been a great benefit…. I can’t praise the sessions enough basically  P03: You get a little bit of a bond with the people you're doing that with 'cause you're all going through the same thing or a similar kind of thing, … I think you know seeing other people and how they are progressing. It gives you a little bit of motivation to progress yourself. You feel like you're not on your own. Sounds a bit stupid, but you know, sometimes you can feel you're battling this on your own and actually, you’re not.  P07: Everyone was proud of everybody else. I don't think proud is the right word, but do you know, you know everyone was like cheering everybody on, and yeah and they were taking turns  P07: but seeing the actual groups with everybody in it was like, ‘oh wow’, you know you just feel you’re kind of on your own, and then you meet someone with exactly the same and what have you. And I really like that and you know, we've become friends, and it's good and, you know, everyone is really happy when they’re learning. And, so am I.  P01: All I wanted from the NROL sessions for me at that time was the physio sessions, cause that was important to maintain mobility. Now, they did some psychology things, I wasn’t really bothered about them so much  P03: I'll be, to be brutally honest, it was absolutely self-centred. The more sort of physio contact, more exercise contact I can get. I want it  P02: I need to communicate with strangers with what I have planned for the future. Getting back to work, communicating with different people of different backgrounds is what I'm gonna be doing.  P02: It was good, it made me start communicating with other people outside of my bubble. Obviously when I had to quit work, I was barely, I was self-employed. I could barely speak to the customers, … but now I've been interacting with people. It's made me able to interact more with strangers. But like I say, I’d speak to anybody but when it all started, and I stopped speaking to everybody. It’s helping me come round  P13: at the minute I'm actually off work, so it's been quite, in a way it's been quite good for me because I’ve not been at work now since last September, so I'm not really doing anything in my day. … so it's giving me more structure and so I know if I need to do a Team session I’ll be ready for it, in a positive way, because it's you know, giving me the structure in the week, whereas before I wouldn’t be doing much. I would just be doing the same thing I do every day, which is not a lot to be honest. So yeah. If I was at work then it would be different, but I'm not ready to go back to work yet unfortunately, so erm it's at least it's getting me up and getting me like presentable, and ready to do a like a Teams call or whatever I need to do. |
| Self-Efficacy | People gained confidence in delivery method | S01: the first block was quite nervy it to be honest. 'Cause it was new … so, bringing it altogether, a bit unsure, a bit nervous about how patients were going to interact with each other, interact with us how what they thought of the content that we put together. People do have a cognitive impairment, so I was a bit apprehensive about could they actually even tolerate being on this kind of forum. But then, as I've done a few groups, I’m less anxious now.  S08: If I'm being honest, I was probably a bit sceptical at the beginning that we wouldn't be able to do from my point of view: cognitive rehab online. I just couldn't work out in my head how do you do, how do I transfer what I do in a person's home one-to-one and make it patient centered into an online group and I think once I realized actually it wasn't instead of it's enhancing what they already have then that changed my kind of perspective on it.  S08: I think at the beginning, I was a bit sceptical that it wouldn't work and that we wouldn't be able to engage patients, so they wouldn't want to come because of the technological side and people will be a bit shy to meet other people. So I think I did have a lot of reservations that it wouldn't work  S09: I've enjoyed the sort of different platform and the challenges. It's made me adapt as a clinician. I think you're much more aware of the subtleties and the change, purely by observation, as opposed to requiring sort of that tactile feedback. So it’s developed me in that way.  S09: So I think in terms of my observations, I hope I'm a little bit more sort of in tune and and hawk-eyed when it comes to like the subtleties of the way people are moving. I think it’s also allowed me to enjoy awkward silences that little bit more [Laughs], but I can see the benefits. I think I have, obviously gone away and reflected and I do have the habit of possibly talking maybe a little bit too much to my patients, given them too much of a sensory overload or too many cues. So it's allowed me as a clinician to just allow my patient to sit there and explore a certain movement, and be OK with saying nothing for a minute or two at a time.  S12: I think adapting to working in group therapy. I think I used to run a group on the rehab ward, but that's obviously in a different setting. I think adapting group therapy to video therapy is it's a skill to have, um, I'm thinking on your feet when you might not have when you might be in a room that's got no resources in around you. You can't just quickly go and grab stuff. So it's definitely more kind of thinking on your feet if you do need to change things, and because of the environment or kind of the participants that you've got in the group. | Gained confidence and motivation with peer support | P07: it just made me think right, ok I'm gonna do this, right I'm gonna do this as well. And it's made me start cooking.  P07: I would say the whole lot of it to be honest. And actually talking to them and to others. Everything like that. Yeah and, and obviously starting to play the piano again.  P13: I think 'cause everybody is like in the same boat as you, everyone can relate to each other as well. Even though we probably like from different. There's different reasons we are there but we can all kind of relate to each other. So even if you do like, you know for me, I just sometimes can stop mid sentence and completely forget was talking about, and so I worry that you know if I go out somewhere and I was talking to somebody, and they don't understand that I have this issue, they would, but on the group that's not really an issue 'cause I think everybody else is in the same boat. So at least that way I can kind of try to do a bit more then, so that I can test what I can and can’t do if that makes sense?  P13: before I was super confident, I wouldn’t have any problems … I think I'm kind of isolating myself because I know now that I struggle with certain situations, so I kind of don't go out as much, so this is giving me that little kind of bit of confidence to talk to people a bit more. And you know, like so I don't want so I won't shy away so much you know in public and stuff. They've just said it's probably better for me. You know, it's like a stepping stone I guess in a way for me, I think anyway.  P01: before this whole pandemic as well came into it, I don't think many people were using zoom, and we weren't but it has also crept in other areas of our life. So you know like I have zoom Church meetings and things like that now yeah, but still actually, 'cause everybody else sets it up for me. I don't physically know how to go on the Zoom. It's 'cause my husband just does it for me |
| Individual Stage of Change | Has had changes in practice | S08: one of the things that I've changed and I'm trying to kind of encourage the rest of the staff to do the same is to start thinking back from the beginning, would this person benefit from NROL so that right at the beginning of their journey into neuro rehab we can get them on the NROL pathway at the right sort of time cos might need it straight away, some might need it further down the road. And I think it's also making me think differently about the patients that are on NROL that I see, actually, if they're having the brain injury education as a group, I then don't need to do that as a one-to-one, we can touch on it and then use that in therapy but I don't need to do that six week program as a one-to-one so it has definitely changed the way I'm doing things. |  | P01: But you know when it all stopped, you know I was panicking a bit really inside thinking, I'm going to go downhill quickly, right? And who's helping me? You can’t stop it, you know, and you know with these chronic conditions, it's a bit like, you know, especially something like MS, it's a bit like you dying a bit at a time because, you can feel yourself losing bits of your abilities and you’re thinking I've got a bit worse at that. And I've got a bit worse at this. And. You know who can stop it? And you think I've only got myself to rely on, what you know and what can I do? And then if you've got the physio, who can, you know like on a zoom you know do some exercises with you, you feel like you're doing something then that might help. |
| Other Personal Attributes | Being adaptable  Therapist preferences | S03: If you go to a patient with a planned therapy session and it's like you've got an hour planned out, you can guarantee it's not gonna work that way. So you've gotta have options and be thinking on your feet. So that's I think something that we're all pretty good at anyway.  S03: I think it had made them (staff) nervous doing the first one and then they kind of get into the flow of it. I know a few people who've kind of done a session where there was presenting something or some information to the patients, and because it was the first time that they've done anything to do with NROL, the fact that they was presenting in the spotlight was on them, they were nervous, but they got on with it, where I can't really think of anybody that would say ‘absolutely, I do not want to do that’. I've really enjoyed it, but I think I suppose it's like everything else, everybody's got the different personalities. Some people prefer working on the ward. Some people prefer working on the community, so some people are going to prefer to do this. Some people are going to prefer not to do this. |  |  |

| **Inner Setting** | | | | |
| --- | --- | --- | --- | --- |
| **Construct** | **Staff** | **Quotes** | | |
| Structural Characteristics | In theory, one over-arching therapy team but in reality, stroke and neuro not worked closely together and based in different locations  - seeing patients from other pathways  Capacity/ staffing an issue  Rehabilitation research relatively new | S08: I think the thing that's been good is she's wanted it to be a stroke and neuro project, not just one or the other and we've always been looking for opportunities for stroke and neuro to work more closely together with our patient groups, because there's lots of obviously similarities. And I think this is really helped stroke and neuro therapy staff to get to know each other better, work together and have that kind of cross pathway work for our patients. It's not just been for stroke or just neuro, it's been both and I've never treated stroke patients before and in the groups I've had stroke patients. So that's been different.  S05: So on balance, you are seeing four patients minimum in an hour, so it still works out over as a service whole - balanced isn't it, but just pulling between inpatient- community.  S09: if you're not seeing the patients from your own pathway, like I’m a stroke physio at the minute, if I'm not seeing the stroke patients, then the contacts drop and the data drops from a stroke perspective. It’s not that my contacts across the board have dropped, because I'm doing NROL, I'm seeing more patients within that group, but they might not necessarily be stroke patients, and you just have a little bit of a dip.  S09: I think historically we've always been quite separate, erm, stroke being like a branch of neuro and stroke eventually leading into the neuro pathway, after like the six month mark following injury. And the services and what patients can access are slightly different. Bringing those two pathways together will be beneficial for the pathways in general, but also NROL and the patients.  S15: I don't know about neuro because I don't really have the contact with them so much outside of NROL. I mean, one of the things that's been nice about NROL is having more contact with the neuro team actually, it’s broadened my horizons a little bit…. it's been quite a bit of work, I think, for the stroke team just to get a sense of actually what NROL’s for and where it sits and what's being asked of them.  S11: we rotate every six months and I'm going to be moving on to the acute stroke unit and I just don't think I'll be able to have the capacity to run a group on top of that with the amount of patients that come through, but we'll see.  S05: Just capacity wise I think, yeah just the commitment and having the capacity to be able to commit to a certain time.  S05: Near enough by the time the group’s done and I have put all my data on, it's almost the end of the day. So, it is a little bit that it’s seven consecutive Wednesdays with my whole afternoons gone. But then if you think of that from the community side, that's at least four patients that in that one hour you've seen, saved on the travel time, suppose its a balance … so I’m inpatients, so I'm losing the in-patient time, but it's seeing the community patients isn't it? Suppose it’s a balance.  S08: So in terms of time, which was my biggest worry is have we got the time and capacity? Now everything's prepared and done it's just a case of agreeing which staff members are running the session and having that kind of confidence and experience to run it  S13: the capacity in the time is the main issues. I think it's been difficult. Trying to, I suppose keep on top of my daytime job. And I suppose juggling my daytime job and NROL, I think the work they have been weeks where I thought NROL could quite easily take over the rest of my week. And there have been a few times where, you know, it's been like, NROL it's just been my life this week.  S08: We've had a mix of experience, so we've done that on purpose really because we've had staff that are quite new to neuro and stroke that wanted to be involved so they've kind of been linked up with the staff that have had lots of experience so that hopefully they feel supported and they feel they've got that person to learn from.  S03: ELHT does not yet have a strong, a strong research ethos that really facilitates and enables this kind of rehabilitation intervention….I don't think anything in the system facilitates a project like this | | |
| Networks and Communications | Variation in knowledge about NROL  Pandemic changed the way teams functioned/ communicated (virtual meetings). Less informal networks.  Emails often not read- need more active methods of communication | S03: Those of us that have been involved in the NROL process, so from the discussions and the presenting of the groups, I would say yes, we have quite a good understanding of what NROL is. And those that aren't involved in it, I would say no, they don't, and. I've been trying to kind of improve this on the Community team  S11: I struggle to get time to look at emails and things like that. …. So it's a case of prioritizing. Unfortunately, things like NROL, that wasn't significant to me as such until I got involved and just went to the bottom of the list. But I think they did really well. I don't know how they could have done more. I think that's just a lot of NHS challenges of time  S17: There’s specific people that are keystones. So I think within the Trust its(NROL) not widely known about, and I think it's more widely known about within the neuro and the stroke team, and I think there's a difference between how aware the neuro team are to how aware the stroke therapy team are, to all the different classes. And I think definitely if I asked the acute team to tell me what NROL was, I think they would really struggle. But if I ask the community team to tell me what NROL was, I think some of them would be able to tell me what the classes were about. So I think even at service level, there’s a variance in what people know about it.  S04: we haven't really had CPD because of Covid, many, you know, sit down meetings, catch up, get together and things like that so it isn't as prevalent as probably what it was twelve months ago. Everybody has been in a staffing crisis and with Covid, it's been a stressful time. So even just thinking about something else has been a lot for other people.  S05: there might have even been emails and things, but again, you know when you just see a word that you don't think applies to you, you just yeah, there might have been emails that came round explaining or… yeah, maybe it was posters or maybe more of a specific talk in the first instance and that might have helped us recruit more staff and volunteers and things to get involved as well, whereas at the moment it's through word of mouth of kind of ‘come and shadow NROL on Wednesday’ or… that's how the words getting out at the moment – people shadowing, whereas we’re in block three already, I didn’t feel like anyone was aware of it in block one.  S06: I think we're managed by two different clinical services which gets complicated, so the nurses are under a different management structure. So the way that information is cascaded down sometimes can miss the nurses. But you know, we've had a team meeting since and discussed it, and one of the ladies who's done a lot on NROL has shared lots of information with us.  S07: So I think it's just continuing to raise awareness and I think for myself and for the people involved in the group and in the project. I think it's just an open dialogue within the team. So for example, every Wednesday we have our MDT meeting and then we sit down and discuss a patient, and if that person would benefit from NROL, will you put a referral in? And so I think that potentially there are a few people kind of still slipping the net but we are really pushing to try improve that.  S08: the staff that had volunteered to be involved in NROL understood it cos we were all attending working parties on a weekly basis and sharing, kind of our worries, our thoughts, our enthusiasm, our reservations. So we, I think we were all on the same page.  I think, perhaps what we weren't as good at because things were moving very fast and we were trying to get our head round it how do you get the rest of the neuro rehab team and the stroke team staff that weren't directly involved in NROL engaged and understanding what it was. And I think that's because we were so absorbed in trying to get our head down to ourselves that perhaps we didn't do as much as we could have to try and get everybody else on board.  S07: I think it's been a slow burner, but having said that NROL's taken off that fast, … I think any new projects take a while don't they to become part and parcel of everyday thinking and life. So I think we're now talking a lot more in the meetings and we've sent out emails, we've sent out flyers. I've done a piece of work for an OT newsletter, which is going to be across the whole of the OT service, not just with stroke and neuro, so it's kind of finding any opportunity really to flag it up. So I think now we understand it better and we've seen the benefits of it, and it's becoming part and parcel of what we do, I think, yeah, people are now on board with it.  S11: I'd heard in discussion a lot more than I’d sort of read information about it. So then I probably haven't always got the gist of it. …. It was under my radar for a while, but I didn't know much about it. So I had to put some time to actually looking into it because, any sort of NROL email I saw seemed to be really detailed and I thought, “I haven’t time to read that”  S13: if you're in the NROL world, you understand it and you get what's going on, but if you're on the periphery and you only hear bits and pieces or you see us walking around without our NROL t-shirts on, it's still a bit of an enigma and. I do wonder whether that's part of the reason why we don't have many patients referred into the speech therapy groups, is that because there is that lack of knowledge out there  S13: they've been roping them (other staff) in, so to speak, and saying, right, come and see what we do in the group. Come and see what NROL is. And by seeing it helps to maybe dispel some of those perceptions or those myths and helps to clarify what NROL is as well, so I think I don't think we could have done necessarily anything different. Like I said, we've been spreading the word. We've been given these explanations as to what NROL is. So I don't think we could have done anything more, really anything different.  P06: I think it's been a very inclusive project and you don't always get that. You sometimes get the bit oh that's my bit stay away. But no, it's been very inclusive and I think very transparent as well. Yeah, we've hit roadblocks on the way, but I think everybody's worked with the road blocks and we've all been understanding from a client perspective from a therapist perspective cause they're having to work outside their comfort zones. And from a, you know the project perspective slash research. I think we've all worked well together. | | |
|  | **Staff** | **Quotes** | **Patient** | **Quotes** |
| Culture | Definition of ‘core business’ of rehab in NHS versus nice-to-have | S03: we have to be careful that not just the kind of the icing on the cake therapy, that they are actually increasing the intensity of the overall offer.  But then I think it comes back to what's our core business, and are we delivering our core business and is this very nice added extra, or actually is this part of our core business and increasing our intensity?  S08: think it's always that dilemma in the NHS isn't it? When are we providing something that's, is it just a social get together and is it part of what we do or should that be part of like another third sector and private or charitable organization or it, should it be part of us? And if, if they're still getting together on a social basis, well, actually rehab's come to an end and we want to discharge them. How do we manage those patients that are still in those groups? And that's the challenge is, you know, cos they could be meeting for the next 12 months, five years couldn't they? But actually rehab is not going to carry on that long  S17: I think that we've probably seen a difference with different professions, actually, in the way they've responded to that, 'cause I think it's certainly from looking at it as a team, the remote consultations were more challenging for some professions than they were for others. So I think from a Physio point of view the idea of doing remote consultations is really quite challenging, 'cause we're such a hands on profession, whereas the speech and language therapists doing it, and actually a lot of what they were doing works really well remotely.  S17: there was a difference in how we approached treating patients during the COVID pandemic. So as a stroke therapy team, the visits were still considered to be urgent, so we worked throughout the pandemic, we still offered face to face sessions for most of our patients, and so although we had our remote therapy facility, quite quickly, we recommenced face to face community visits. There was the obvious exceptions with nursing homes not allowing people in. So I think as a team, we never we never stopped delivering our core business, whereas the neuro team just 'cause of the nature of their patients, they did stop visiting. So they, I guess they had more of a need to find an alternative way to deliver a service. And possibly more time to be aware of it as well, and more time for… I think there's an imbalance between how many stroke professionals and how many neuro professionals are involved with NROL, so probably more time to actually attend meetings, develop services and provide those classes as well. So I think then information is probably been disseminated just a little bit more as part and parcel through the neuro team. Through the stroke team as well, it's a bigger team. We have more turnover so I think we're probably just not instilled that process of making NROL a part of what we talk about on induction. It’s getting better. We're more aware of it, and there's definitely in meetings we have an NROL section where people do updates, and in the MDT meeting we have a section where we have prompts and you know – would they be suitable for NROL? So it's definitely becoming more a part of what we do.  S02: And I think the other thing we've got to be mindful of is that to deliver this is requiring a culture shift and a mindset shift in the therapists, and they're not all ready for that yet. So we've only had a few groups running. There's resistance in some places, and there's absolutely enthusiasm in others. So at the moment we’re not being led by necessarily what the patient's need, we’re being led by what therapists want, and that's fine at this point. But we have to get to the point where we know what the suite of interventions are that work well for this methodology, and how we're going to deliver them.  S15: So I think it's just trying to sort of weave NROL into the daily fabric of life rather than feeling like a separate thing. So I think we're getting there, but it’s slow.  S07: I think we're getting a mixed bag to be honest. We're getting a lot of staff that feel it's worthwhile. A lot of patients that feel it's worthwhile as well, and a lot of really positive feedback from the staff that are doing the sessions. And they’re obviously feeding the patient feedback through to us as well. Erm, there is a percentage of staff that just questioned the longevity of it.  S14: Because a lot of the time they'll prescribe therapy services, as not, we don't provide maintenance, we provide a block of treatment to improve a patient and then discharge them. But then do we need to actually relook at the model in which we’re treating patients, because if we maintain them then they are less likely to deteriorate, therefore the cost of living and all that sort of costs are going to reduce, cost of carers that sort of thing. But I think unfortunately the NHS looks at the acute side of things in a short period of time, and the amount of money they can save there rather than looking at longevity. If we keep people physically active by doing groups, therefore they'll stay more mobile, they'll reduce the falls risk. They won’t fall and break the hip, cost all the extra cost to the NHS, have to then have carer’s costs, social services, all that sort of thing. And actually, by doing this, does this actually keep these patients more active, more independent and less dependent on the NHS and social services?  S02: we definitely did bring more group therapy than we would normally do face to face. So we have groups running now that would not be the norm. | Patients not aware of Teams and used to using other platforms- understood NHS restrictions  Patient concerns about therapists referring to groups and not patients choosing their own groups | P06: I think the problem is to do with the confidentiality and I think it's the health authorities rules on the platforms that they use etc…. They won't use Zoom. I think it's a bit outdated.  P06: what does concern me is the feedback that the therapist is going to decide who goes on what …They don't know what they don't know. Because we might have chosen not to reveal that. For whatever reason, we deemed it inappropriate that they know they don't necessarily have access to everything or a right of access to everything. And its only when we want to bring that into the arena to say, well, yes, I do struggle with that. I think I'll give you a good example. I disclose that I have difficulty sometimes in returning information and sometimes I'll struggle with the word. I'm very articulate, so I can usually get round it. I spoke a lot today, hence I couldn't think of a different word, but I have techniques to do it, right. I chose to disclose that and as a result of that I ended up on the ‘tip of the tongue’ group. But if I hadn’t have disclosed that, would I have ended up on that session? And I derived a lot of benefit from that.  P06: I think what they're trying to do is control more, who goes on it rather than you select I want to go on it. The therapist wants to select who, go on it, which seems appropriate. It's a medical model after all. It's not about what interests you the most, etc. But, I suppose it's like everything else. If you don't know that there is an opportunity to gain more insight and more knowledge, how do you know that that's not appropriate from a client perspective, but also from a therapist perspective, you know a facet of a client you don't know everything. |
| Implementation Climate: *Compatibility* | Combining 2 services  - different ‘fit’ for each  Challenge of limited capacity in NHS  - NROL could help with waiting lists  - audit requirements  - delivered by staff at home due to shielding/ isolating  Another part of the rehabilitation process and pathway, considering the fit going forwards | S02: combined two services to deliver this, and they're not natural, they’ve not been naturally combined before, so so we're having some quite significant changes in service modeling,  S03: The difference comes when you're mixing acquired and progressive. So that's gonna be a challenge, and I think the other challenge, which I don't think we know, is the difference between patients who have intervention early in the pathway and patients who have it later in the pathway. And I don't know if we know for the different presentations what the right time is for NROL, and it could be that for acquired brain injury it's early post-discharge and for stroke it's late post-discharge. We don't, but I don't think we know that. So that's got to be one of our outputs.  S14: And I think there has been a bit of a challenge with the acute versus the progressive condition, and how you can match them up, because, does it impact on patients with progressive conditions when they see an acute patient that has a stroke and that's it, and they improve. Compared to somebody that has MS, or a progressive condition, that they have the diagnosis and they constantly deteriorate over time. So kind of seeing them two perspectives - ‘Oh, well, that patient is improving every week, but I'm staying the same or actually I'm getting a little bit worse’. They still see some, at lot of them do still see improvements, but they may not see it to the same as the other ones. And how does that work? Do we need to separate them? Or actually is it beneficial for them to both be in the same group to kind of share different experiences? So I think that is one of the challenges.  S08: it's been something we've been trying to find ways of doing better and never really, I don't think we've ever really had any real projects that have really involved cross pathway working with therapy staff in one pathway, working with the patients from the other. There isn't anything else that has done that.  S14: I feel like neuro has been the main driver because we have a lot of patients, and we have a longer waiting list and we have reduced staffing. So there is a lot of pressures there for us, and we see group therapy as a good thing for some of our patients, cause they do have longer term conditions that need sort of pushing towards self-management.  S03: NROL could be a way of reducing those waiting list… but I think we're in a situation where the therapists are so busy they can't see the wood from the trees.  S14: I think the stroke team had issues with staffing and capacity … I think one of the things they've missed a trick with is the fact that they can include NROL in the SSNAP audit numbers. And actually if we can get that across to them, then they might be able to see it as a benefit of -maybe replacing one of their sessions a week with NROL might be helpful. Or on top of that, it might be an additional session.  S09: there was another member of staff who fit the role a little bit better. She was going on maternity leave. So it kind of made more sense for her to do that, to run that group … because it fitted in with her case load and her diary, and working from home.  S02: Some just don't wanna do it. Some people don't want the tech. Some people don't want to change their practice. Some people say it's too much hassle, and they can just go along with what they know. And so I think there's a whole raft of reasons, some of which are acceptable and some of which aren't. So we know that you know some therapists will naturally be better in certain environments, and you should always play to a therapist’s strengths in that sense. But by the same token, if it's decided that this is a model of service delivery, you can't pick and choose. So whether we decide this is how we deliver intervention, then everyone will have to be signed up to it because we will have to make sure that all patients are having equal access, and all therapists are equally supporting it, even if they don't all equally deliver it.  S15: I knew the model from what UCL had done and I knew that they had very specific focus psychology groups around stroke with the sort of look into the adjustment kind of thing. .. So I had a sort of the vague sense that it would be something like that. But I was again thinking, I don't think I'm going to be able to do it as they did at UCL because they had five days a week psychology to do NROL. And I've got two days a week psychology to do NROL alongside all the other stuff with my job that I try to squash in two days a week. So I kind of knew it was going to have to look a bit different. But I don't I don't think I had much of a sense of exactly what it was going to look like.  S15: the way it's set up at the moment, it doesn't save me clinical time. So, the people coming to my group, it doesn't save me like six one-to-one sessions because the majority of them are neuro patients, so I won't be seeing anyway. And then the stroke ones they’re sometimes people that I don't think would have been referred to me, for pure one-to-one therapy. So I think it's an interesting thing to do and it's an important layer of people's rehab. But I think that it's the time. It is extra time that it takes me. Basically, you could do more if you have more time.  S03: I know that a lot of us kind of try and get that over to patients and the family that like the most important therapist is themselves - Yes, you've got your experience physio your experienced occupational therapist coming in to see you to give you advice. But if you're not your own therapist in between, then you're not really going to get anywhere. ….. So it's very much part of the rehab process anyway  S04: stroke in particular, although it's come about because of the pandemic. We were still seeing patients at home….They have been having face-to-face therapy, so I was wondering about how it would fit in, but we're very aware that it's not instead of it's alongside and it can be helped along with face-to-face. So, it's not taking over anything.  S07: And I think what we're finding is that there's not necessarily a particular point in the pathway or a particular point in the journey where NROL is suitable, I think every patient is different and I think we just need to look at it on an individual basis really.  what we do need to kind of bear in mind is, for the patients in this who are under the stroke team that are involved in NROL, it isn't just NROL that they are receiving. So yes, NROL is a significant proportion of the therapy, but they will also be having face to face-to-face contacts as well. So I think any improvement would be a combination of the two.  S08: I think NROL has got a place to play in addition to one-to-ones. I think it's just working out whether the way we formatted it is the right format or whether it needs rejigging. I think we're still trying to establish that ourselves, but I think there's definitely a role to play in Neuro Rehab online, but not as 'an instead of '. It's kind of enhancing and providing that extra rather than instead of. | Challenge of fitting in NROL with routine  Compatible for long term conditions | P06: I’ve had to prioritize it. .. it's the concept sometimes that people think you've got nothing else to do because you're ill… like we were starting doing cafe NROL at eleven …Why is it eleven? Who decided eleven? Eleven cuts off you’re day, most of the people are morning people. Some people were saying that they got into work activities so they forgot the cafe NROL and I said as Covid starts opening more things when we get into better weather. We're going to struggle now it's been put at ten o’ clock, but I don’t know who decided ten o’clock, I'll be honest I think a therapist decided it was ten o’clock and ten o’ clock It is. They weren't like a lot of clients, whatever, but I'm not gonna moan about it … quite a lot would have liked them at nine. So nine until ten, It is the first thing that you do. You don't forget it. So you increase the attendance rate for start up from a therapists point of view, actually, so you just block it off that day. It's done, and then you're free for the rest of the day  P06: they did nine in the morning for speech and language which was brilliant for all of us. Set you up well, we've done it. It was done, out of the way. We could practice it for the rest of the day and it was great, You know. Physios we did in the afternoon, that did clash with something had to drop off a college course because they changed the college times, it didn't clash at the beginning, but then it did. But had to miss the last hour of college to do the physio. So happy to do that. cause I got benefit from it.  I can see as a therapeutic model, that it’s viable going through from somebody who's been in rehab a long time and as a long term illness, it's never gonna go away. These are wonderful opportunities to have, kind of refresher bits where you don’t tie up therapeutic time. |
|  | **Staff** | **Quotes** | | |
| Implementation Climate: *Learning Climate* | Co-creation, safe and reflective | S01: everybody talks to each other. If we find a problem will let each other know what that problem is and how to overcome it. And that's where I come in as well when I'm online. If I see someone struggling with something, I will provide them with some help, how to get over that problem and hopefully they'll learn from that, and then they will pass that on to people that they have with them as well. They may have trainees with them. And all the other staff with them that they will then pass that information onto as well. So, everybody helps each other out.  S04: It is reassuring and I do feel like I’ve learnt, and I’ve showed other people little bits of things as well  S05: Definitely people are interested in hearing about it, but I think you can tell the difference between people who you would consider to be able to be involved one day, … rather than the people who are like ‘How's it going?’ But they've got no inclination of getting involved. But no I wouldn't say we faced any resistance.  S07: I know particularly one of our band sixes has actually really enjoyed developing a group and having the opportunity to kind of see that into fruition.  S08: We've had a mix of experience, so we've done that on purpose really because we've had staff that are quite new to neuro and stroke that wanted to be involved so they've kind of been linked up with the staff that have had lots of experience so that hopefully they feel supported and they feel they've got that person to learn from. So, yeah, I think we've had a mix of experience, but we've dovetailed people together to try and balance that time  S09: I think I heard something the other day that we are looking at starting the face-to-face groups again, so there's always going to be a little bit of competition now with who we refer into which group. Do we sort of prioritize NROL, do we prioritize the face-the-face groups? So, I think that will be an interesting battle between the two, and particularly with who refers in, as a clinician - who refers in to which group, and who prioritizes which group over the other.  S11: we probably worked a little bit more collaboratively than I would have done if I was just seeing someone on my own. So we did some inferencing and I've never even really come across that much and we did a whole section on that, so it was very speech and language focused. Which was good for my learning as I got to know all about that in that session as well. But it's not something that would’ve normally touched on.  S13: I've learned from doing rather than kind of prepping to deliver if that makes sense. I've learned kind of from a first block I’ve thought. What went well, what didn't go so well. And use that to kind of influence how I do my second block of groups, whereas I think if I'd had more time at the beginning, I don't think I would have envisaged every single thing or would have planned for something that probably wouldn't have happened | | |
| *Relative Priority* | Other demands and system changes happening at the same time- competing priorities | S07: We implemented an early intervention service, and so our KPIs required us to be quite responsive… and I think sometimes from a staffing point of view we've had to pull back and say this is what I feel able to provide, and it's maybe not always been as much as potentially was wished for or was needed at sometimes, but very much feel like NROL has its place and there are a lot of benefits to NROL, but I think as a team we need to make sure that we're not diluting our service for our kind of face to face contact as well.  S07: as a team ..we were struggling to recruit stroke patients into the group, so I think in the very early days we were providing a member of staff to run the group, but actually didn't have very many stroke patients. Erm, and kind of from a capacity in the team point of view it didn't at that point feel the most efficient way to do things  S03 it is a completely new way of working. Which is great. But then if you’ve got twenty odd people on your waiting list and you're trying to plough through them because they’re breaching and stuff like, that to find time to sit down and absorb what some things about NROL, it's a case of priorities isn’t it. And I don't know what the priorities are.  S03: you always think I've got this many people to see you? I've got this many amount of referrals. And yes, in the long run this might potentially help that, but I've gotta put these many hours in to get it set up and I don't have time.  S04: Everybody has been in a staffing crisis and the Covid, and it's been a stressful time. So even just thinking about something else has been a lot for other people  S15: I think some people have just felt like they haven't got time for new and complicated things right now, so they’ll just ignore it, thank you very much, because alongside NROL, I guess we were also relatively new, the setting up of early intervention service in stroke and just all the general disruption of Covid life, and we’ve had quite a lot of major staff changes as well. So I think for a lot of people, it just felt like there was too much going on. And just keeping the day-to-day stuff that we knew of community up and running and ticking over was enough. So I think some people are just for their own sanity, chosen to just ignore it for now. But it would be nice to kind of bring them in as the dust settles in the rest of life, if you know what I mean.  S14: it was a no brainer really. We needed to start something.  S17: I think you have you got you had a a workforce who were still coming into work every day during COVID and going out to see patients or working on the wards, seeing patients, dealing with the whole PPE. Treating COVID patients, knowing what to wear, knowing not what to wear, doing lateral flow test to get in some places. So I think we've had a year of being bombarded with guidelines about what to wear, how to approach things and they have changed frequently, and we've all had to change our practices during the last year. And it's been a year of kind of stress at work and outside of work for many reasons for people. So I think I just think headspace, oh and also yeah, massively for the stroke therapy team, during COVID they've actually started a new service, so we've started the early intervention team. That launched, I think just as the COVID pandemic started, and it didn't not launch because of COVID. So the team have developed this new way of working, new processes new… they've expanded, so there's just been a mass of things going on, so I think people have just not had the headspace for something else. | | |
| Readiness for Implementation | Clinical academic partnership and SameYou funding a key enabler | S03: because I already have this partnership between the UCLan and ELHT which is [Name] post. We just done it on the back of that, but without that it just wouldn't have happened. I have a feeling that a lot of it would have just gone on the ‘too difficult’ pile.  S03: we’ve been talking about it (groups) for years…but the fact that we’ve had the oomph from UCLan and SameYou, I don’t think without that would have ever got off the floor  S14: The Same You charity. Yeah. And I think if we didn't have that, I don’t think the Trust would have invested in it at that point | | |
| *Access to Knowledge & Information* | Tech support/ UCLan all key  Supported to take the leap and just start | S08: I think now that we've prepared it, all the hard work at the beginning has paid off because now we've got crib sheets, we've got interactive sessions planned, we've got all the kind of materials and resources are done so that was the hard bit at the beginning  S14: I think it's been well supported once we could evidence the impact of it. And we've had a lot more engagement sort of in the last few months when we've been doing presenting the results and some of the outcomes, and people seem really engaged by it. I think we're lucky in a way, I think we wouldn't have got it off the floor if we didn't have the charity funding. I don't think we would have got it anywhere near to what it is. | | |
| *Available Resources* | Space and technology challenges in NHS | S05: hot desking and kind of dashing around for a laptop that works half an hour before doesn't work and then Teams isn't loading and it needs updating... that wouldn't be sustainable to do.  S05: like you might have heard - four people have walked into the conference room where I am now, in the 50 minutes we’ve been on the call. We've got a couple of offices so obviously, not private areas. We've got one quiet room at (the unit). That's technically a patient room for quiet therapy, and so we generally put signs on the door… And yes we kind of just try and hope that this room is free for anything.  S08: we've got several laptops that we've got access to, but if there are other staff having meetings at the same time, then it's how do you share all the technology out? So that sometimes has been an issue trying to prioritize. We've also found easier at the beginning to have two staff in the same room on, on a different laptop so that we can kind of communicate to each other with our sort of eyes, who's when leading the session or if we can see something's happening, we can kind of communicate better to each other without interrupting the actual session. So trying to find a room that's big enough for social distancing and not having to wear a mask, which then isn't ideal onscreen, that's been a bit of a challenge. And I think going forward we thought, once we get a bit of experience, using one person in a session might be the way to go, but having two screens where you can share a screen and still see the patients on it would be beneficial as well. So I think that would be a bit of: how would you set that up? Where would you do it? Which rooms will be available and have we got enough technology to do that?  S09: Sort of, the facilities and equipment, and space really, for the lower limb, … a lot of it is done in standing, a lot of ways sort of moving about. A lot of the exercise is quite active. So, you need a lot of space...at the minute we've only got the gym with the equipment set up as well, with the sound, the laptops and the technology. And with staff being kind of here, there and everywhere, branched over about three or four sites, trying to coordinate diaries and get people to where they need to be can a little bit difficult. | | |
| *Leadership Engagement* | Commitment from all areas of leadership- clinical, managerial and research | S02: We've had sign up from Physio, OT and speech therapy, so I think we know who our champions are, and I know behind them there are a group of other staff who want to get involved, and a group of other staff who don't want to get involved. We've had to do some chivvying along, to get people out of their comfort zone and actually get up and running with it.  S01: I mean the people behind it. You've got the therapist themselves, which do an absolutely wonderful job. They’re amazing at it. But you've got people like (clinical academica), who worked tirelessly behind the scenes to try and pull everything together and make sure everything runs smoothly as possible. You know there are real workhorses behind this, and without them, none of this would ever have occurred.  S02: what's really important as the service manager - I'm really keen that NROL is one of the tools in our kit.  S03: (Name) [laughs], quite simply. Because she was, she had a deadline and she was on it to get everybody doing what she needed them to do by when she needed them to do it.  S03: our top manager is very into it, because in the long run it's you know it is saving time…I would say like all the managers are very much for it, and some of them are involved in it, like some of the band sevens, the band eights are actually involved in it themselves anyway  S07: So I think obviously [Name] initially did a lot of the communicating, and a lot of the pushing forward, and I think her and her team are definitely instrumental into the kind of the building up of it  S07:l I know particularly erm kind of my operational lead, and service lead as well, has been really positive and really pushing NROL. She really sees the benefit, so we have been supported from that perspective. Yes, definitely  S11: (Name) sent a few e-mails around with lots of hints and encouraging involvement with it and especially in Cogs in Motion, which is why I initially volunteered for, and then began the opportunity of doing a new group, which was good. So, yes, (name) was very, just opened the opportunity I suppose. And she thinks a lot of it and values it, and she wants it to be successful and continue | | |

| **Outer Setting** | | |
| --- | --- | --- |
| **Construct** | **Staff** | **Quotes** |
| Patient Needs and Resources |  | S02: I think patients will overtime get more used to it because more stuff will be done remotely. And you know, we have to think about the next generation of patients and therapists. For whom this becomes the norm, rather than you know, some people like me for whom it's a massive change  S14: there's a massive push, isn't there, for digital inclusion and we shouldn’t be excluding people because they don't have the technology or the skills to access the technology. And I think that's a really big thing that we need to continue to look at. And I think with the charity they are looking at sort of ability for patients to access devices and that sort of thing, so it doesn't exclude them  S16: I was thinking, just because our client group often are older, they might be less tech savvy. So I think that was the worry. But obviously, there's been a lot of tech support, so that's not been such an issue for people to participate.  S01: So, there's always going to be a demographic out there that won't want to associate with this type of therapy. And you can always imagine that not everything is going to be wanted by everybody. Everybody's needs are going to be different. Everybody's characters are different, but yeah, wherever a person's character would allow them to do something like this. I think they'll get great benefit from.  S12: I also think a lot of people don't want to take part in group sessions, we've had a few people saying that that's one of the reasons they just don't want to be in that kind of group situation, I guess is it's not for everyone, is it?  S08: I think some people are very, very much up for it love it, turned up earlier every session and really get a lot out of and then I think there's the others that aren't as committed and they'll turn up a bit more hit or miss, but then I think that's the same with, if you think, if you look at the patients that hits and miss, they're probably hit and miss with the one-to-ones as well. Cos it's not, it's still the same sometimes you'll ring up, "I'm on my way. Am I still okay to come?" "Oh, I forgot you were coming." You know, "I'm not in at the moment. Can we rearrange?" So I think, I think you probably gonna get that anyway. We still get it with one-to-ones.  S11: it was seen as an added bonus, … I don't think she held it at the same value as another appointment, as it was just an added thing that she'd not go to if she had anything else on. |
| External Policies and Incentives | Covid restrictions and context constantly changing | S02: at the moment who's supporting them could well be restricted by Covid restrictions. So we can't send a volunteer in to support them  S08: during lockdown patients haven't been able to go anywhere so there've been available … I wonder whether as people are allowed to go out and meet and do other activities, whether they will be as committed to come into the sessions and whether other things will take priority in their life, rather than, whether they will prioritize NROL as, as part of that rehab.  S08: during COVID we knew that patients weren't going out, they were very isolated. They weren't able to access the same things as normal to actually give themselves an opportunity to improve the everyday, whether it's communication or put their cognitive skills into practice or you know, meet people, they weren't having that opportunity. So I think having online sessions was a way of giving them that opportunity to see other people, support each other and yeah, and I don't know, not feel as isolated I guess, as they, a lot of patients where I think a lot of my patients feeling quite low in mood so I was hoping that was a way of trying to give them some motivation and lift it a little bit.  S11: I think the families have been really supportive with it all. And I think because a lot of them have been furloughed and things at the moment, that's worked well.  S13: at the minute we've kind of almost got a captive audience, everyone's staying at home, so they've got the opportunity, the availability to log on to a group, whereas if they are also going out and about more and maybe socializing or maybe accessing other groups, like, for example, the strokes association, they run groups and they may be going to those groups rather than our groups. So I think it would depend on their availability, but equally, I think it would also depend on their rehab need, because if they still need rehab at the end of the day and this is something, this is a way that we're offering rehab, then it's almost like, well, do they how much do they want it? And see, I think it would depend on those things, I don't think it would. I can't see it changing from our point of view, I just see it changing from a patient point of view.  S12: I think NROL is an opportunity for patients to get therapy from within their own home whilst remaining, say, shielded. It is an opportunity for them to continue their rehab within the pandemic environment where we're trying to reduce footfall into people's houses, reduce the risk of spreading the Coronavirus. I think it's an exciting opportunity, … it's interesting because it's something that maybe we've had the tools for a while, but there had to be that shift of something that we couldn't go into patients houses or we couldn't do group therapy. So we had to think about this new opportunity that we could give patients for them to continue the opportunities that they would have had without the pandemic I think  S12: patients weren't going out and seeing friends and family again. So it was it kind of came at the right point of the third wave or whatever it was. So, yeah, I think it's been really helpful.  S14: It's almost like the lockdown has shocked everybody into thinking in the virtual world a lot sooner than we would have  S17: it's been a needs-must kind of reaction. …. Yeah, definitely triggered by the COVID pandemic and definitely challenged our thoughts about the whole remote process. |
